# Clinical assays rapidly predict bacterial susceptibility to monoclonal antibody therapy

**DOI:** 10.1172/jci.insight.174799

**Published:** 2024-01-23

**Authors:** Matthew J. Slarve, Neven Bowler, Elizabeth Burk, Jun Yan, Ulrike Carlino-MacDonald, Thomas A. Russo, Brian M. Luna, Brad Spellberg

**Affiliations:** 1 Department of Molecular Microbiology and Immunology, University of Southern California, Los Angeles, California, USA.; 2 Department of Medicine, Veterans Administration Western New York Healthcare System and University at Buffalo, State University of New York, Buffalo, New York, USA.; 3 Los Angeles General Medical Center, Los Angeles, California, USA.

**Keywords:** Infectious disease, Bacterial infections

## Abstract

With antimicrobial resistance (AMR) emerging as a major threat to global health, monoclonal antibodies (MAbs) have become a promising means to combat difficult-to-treat AMR infections. Unfortunately, in contrast with standard antimicrobials, for which there are well-validated clinical laboratory methodologies to determine whether an infecting pathogen is susceptible or resistant to a specific antimicrobial drug, no assays have been described that can inform clinical investigators or clinicians regarding the clinical efficacy of a MAb against a specific pathogenic strain. Using *Acinetobacter baumannii* as a model organism, we established and validated 2 facile clinical susceptibility assays, which used flow cytometry and latex bead agglutination, to determine susceptibility (predicting in vivo efficacy) or resistance (predicting in vivo failure) of 1 newly established and 3 previously described anti–*A*. *baumannii* MAbs. These simple assays exhibited impressive sensitivity, specificity, and reproducibility, with clear susceptibility breakpoints that predicted the in vivo outcomes in our preclinical model with excellent fidelity. These MAb susceptibility assays have the potential to enable and facilitate clinical development and deployment of MAbs that generally target the surface of microbes.

## Abstract 2

<p>Rapid, antimicrobial susceptibility tests that can be deployed in clinical microbiology laboratories to support monoclonal antibody therapy for infections.</p>

## Introduction

The emergence of antibiotic resistance is a key threat to human health, fueled by overuse of broad-spectrum antibiotics, and is estimated to cause 23,000 deaths annually in the United States alone (1). Given that each use of antibiotics contributes to selective pressure driving resistance, one potential alternative strategy to treating infections is to use immune-based strategies, such as monoclonal antibody (MAb) therapy, which may be less prone to inducing resistance due to their activation of multiple mechanisms of protection simultaneously (2–6). Indeed numerous MAbs have been developed and entered clinical trials in the last 15 years to treat infections caused by bacterial or other pathogens (7–18). However, one limitation of all such MAb therapies to date, which has made clinical development and future clinical deployment more complex, is the lack of a rapid in vitro “susceptibility” test to determine if a specific MAb is likely to be effective in treating infection caused by a specific pathogenic strain. In contrast, antimicrobial susceptibility testing for antibacterial and antifungal small molecule agents is routinely done in clinical microbiology laboratories, informing treating clinicians regarding which antimicrobial agents are likely to be effective for treating a specific infecting pathogen.

We have developed numerous MAbs with preclinical efficacy targeting the highly drug-resistant pathogen *Acinetobacter baumannii*, including MAbC8, MAb65, MAb5 — and 1 bispecific MAb — BsAb C73 (6, 19–22). Additionally, we introduce here a MAb called MAb10, which complements the binding breadth of our previous MAbs. To support planned clinical development of these and other MAbs, we sought to establish a methodology to enable a rapid, high-throughput test for MAbs that would identify if a given pathogenic strain was likely to be effectively treated by the MAb, equivalent to standard susceptibility testing of antibiotics used in clinical microbiology laboratories. Here, we demonstrate that 2 facile, high-throughput assays designed for feasibility in a clinical microbiology laboratory accurately predict in vivo efficacy preclinically. Such assays will enable clinical development of the MAbs, and if validated in future clinical trials, will support greatly expanded development and clinical deployment of MAb therapies targeting a variety of prokaryotic and eukaryotic pathogens in the future.

## Results

### MAb10 exhibits broad binding of A. baumannii strains.

**Figure 1:**
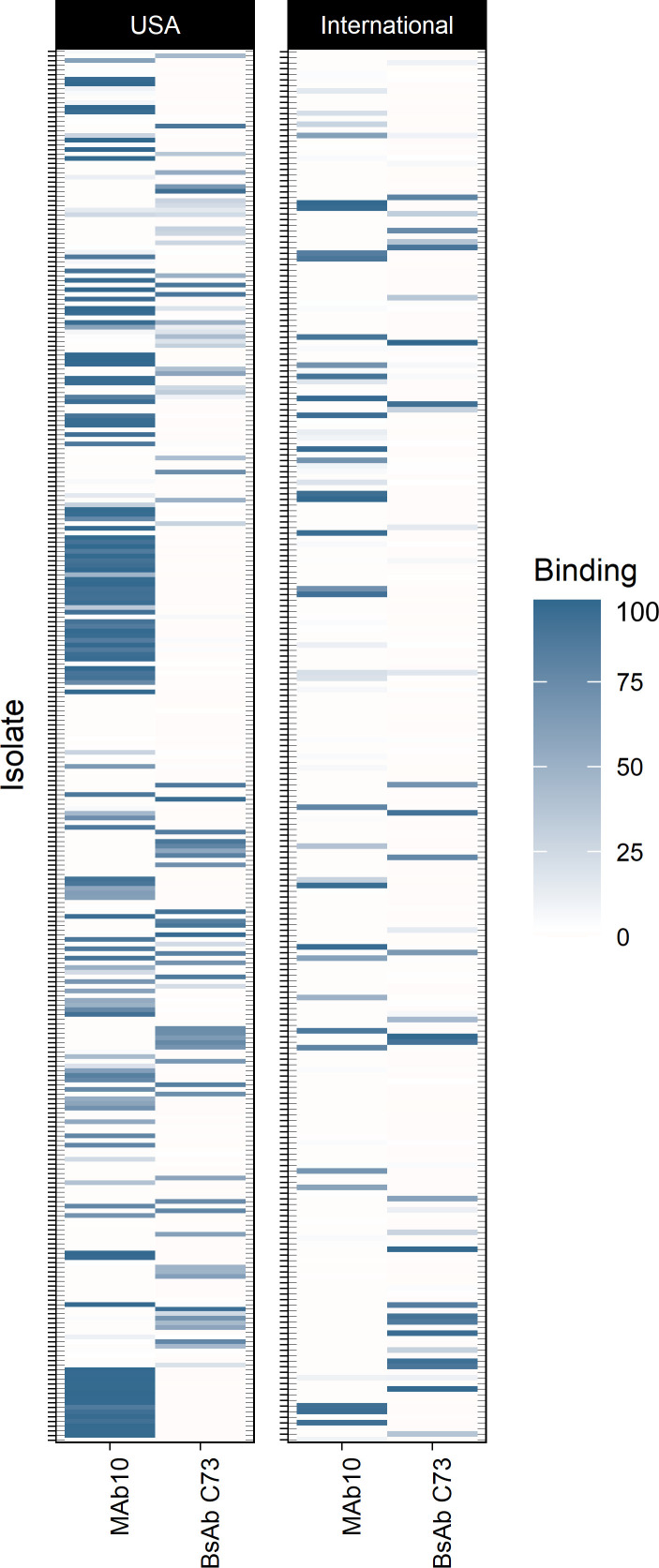
MAb10 exhibits broad binding of US and international *A*. *baumannii* isolates. A panel of strains from hospitals in the United States (*n* = 300) and a panel from international hospitals (*n* = 250) were assayed for binding with MAb10 and BsAb C73 via flow cytometry. Binding is observed in 151/300 strains from the United States and 72/250 international strains. Coloration represents the percentage binding per isolate, defined as the percentage of events in the MAb10-treated group of the flow cytometry assay that were brighter than the control group. The full explanation of the flow cytometry gating strategy, and how percentage binding is calculated, is included in Supplemental Figure 1.

We previously produced MAbC8, MAb65, a resulting bispecific BsAb C73 combining C8 and 65, and MAb5, all of which bind to the surface/capsular polysaccharides of *A*. *baumannii*. These MAbs have impressive specificity and in vivo efficacy (6, 19–22), and when we screened against a library of 550 clinical *A*. *baumannii* isolates sourced from the United States and abroad, we found that these MAbs combined bound to a total of 59.85% of strains with nonredundant coverage by standard flow cytometry immunofluorescence (Table 1). To expand this binding breadth, we sought to produce an additional MAb by immunizing mice with a mixture of 30 strains against which none of our prior MAbs bound. Immunization and boosting led to the generation of a hybridoma cell line producing an IgG2a monoclonal antibody named MAb10, which had binding against 151 of 300 *A*. *baumannii* strains from the United States, and 72 of 250 strains from international sources, as assayed via flow cytometry (Figure 1).

**Figure 2:**
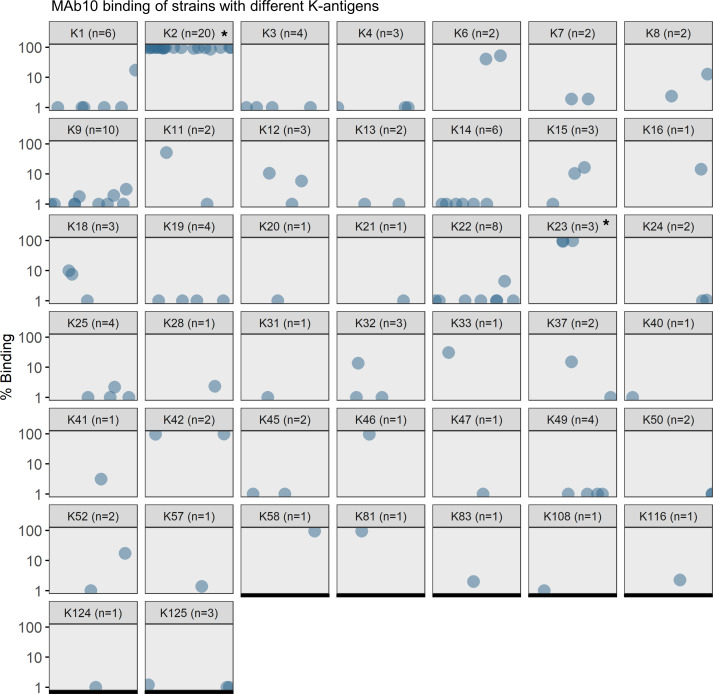
MAb10 has clear specificity for specific K-antigens. A panel of strains (*n* = 123) with known K-antigens was assayed for binding with MAb10 via flow cytometry. Percentage binding is shown on the *y* axis, with different strains on the *x* axis. Each subplot represents a different K-antigen type 1–125. Given that each strain has only 1 K-antigen, each strain only appears in 1 subplot above. Binding is elevated in K2 and K23 over other K-antigens (*P < 0.0001, 1-way ANOVA). K-antigens K6, K42, K46, K58, and K81 likely also have elevated binding, but too few strains with those K-antigens were available for thorough statistical analysis.

MAb10 was also screened for binding against a panel of isolates (*n* = 123) with established genomic sequences (Figure 2). Using a local Python-based version of the tool Kaptive (23, 24), we analyzed these sequences to determine the strains’ K-antigens (the polysaccharide chains that make up the capsule). Stratifying MAb10 binding data by the different K-antigens demonstrated that MAb10 had strong binding to K2 and K23 capsule types (*P* < 0.0001, 1-way ANOVA). Though few strains of these types were available for screening, MAb10 also appeared to bind K6, K33, K42, K46, K58, and K81. The addition of MAb10 to our MAb cocktail improved the overall binding from 59.85% to 72.45% of strains (Table 1).

### MAb10 exhibits protective efficacy against A. baumannii.

**Figure 3:**
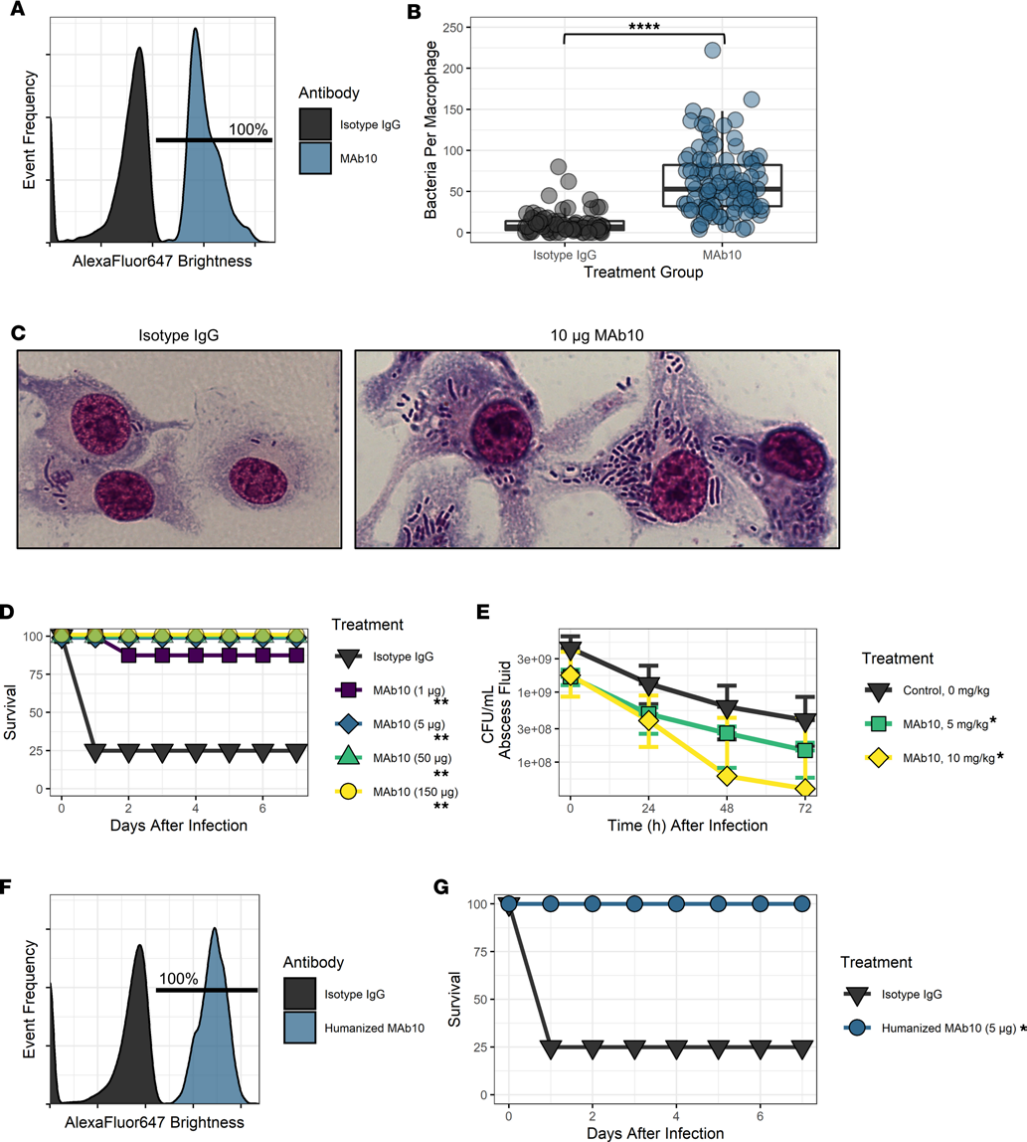
MAb10 has potent efficacy in in vitro and in vivo assays. (**A**) *A*. *baumannii* strain 1127417 was incubated with murine MAb10 (blue peak) or isotype control antibody (black peak), followed by an Alexa Fluor 647 secondary antibody, and 10,000 total events were assayed via flow cytometry. The gate shows the percentage of events in the murine MAb10 group that were brighter than the isotype group. (**B**) IFN-γ–activated RAW 246.7 cells were infected with murine *A*. *baumannii* strain 1127417 and given either isotype control treatment or 10 μg murine MAb10 for a 1-hour incubation. Cells were fixed and stained with Hema stain, and phagocytosed bacteria were counted in replicates of 5 photos per well and 3 wells per treatment. ****P < 0.0001, Mann-Whitney *U* test. (**C**) A 100× light microscopy photo with oil immersion showing RAW 246.7 cells having phagocytosed *A*. *baumannii* 1127417 with either isotype control treatment (left) or 10 μg murine MAb10 treatment (right). (**D**) C3H mice (*n* = 8) were infected with 4 × 10^7^ CFU/mouse *A*. *baumannii* strain 1127417, then given either isotype control treatment or 1, 5, 50, or 150 μg murine MAb10 treatment. **P < 0.01, log-rank test. (**E**) Sprague-Dawley rats (*n* = 4) in which a subcutaneous fluid collection was created were infected with 1 × 10^9^ CFU/mL *A*. *baumannii* strain 1127417, followed by intraperitoneal delivery of 0, 5, or 10 mg/kg MAb10. Fluid aliquots were collected from the abscess at 0, 24, 48, and 72 hours after infection and assayed for CFU/mL. *P < 0.05, 1-way ANOVA. (**F**) *A*. *baumannii* strain 1127417 was incubated with humanized MAb10 (blue peak) or isotype control antibody (black peak), followed by an Alexa Fluor 647 secondary antibody, and 10,000 total events were assayed via flow cytometry. Gate shows the percentage of events in the humanized MAb10 group that were brighter than the isotype group. (**G**) C3H mice were infected with 4 × 10^7^ CFU/mouse *A*. *baumannii* strain 1127417 and given either isotype control treatment or 5 μg murine humanized MAb10 treatment (*n* = 8 and *n* = 7, respectively). *P = 0.0112, log-rank test. **D** and **F** represent the summed data of 2 separate in vivo experiments each.

We sought to evaluate the protective efficacy of MAb10 against *A*. *baumannii*. Isolate 1127417 was identified as an *A*. *baumannii* strain against which MAb10 had 100% binding (i.e., 100% of events in the MAb10-treated group in the flow cytometry assay were brighter than the control, Figure 3A; the gating strategy used to determine percentage binding is in Supplemental Figure 1; supplemental material available online with this article; https://doi.org/10.1172/jci.insight.174799DS1). Furthermore, MAb10 enhanced RAW 246.7 macrophage opsonophagocytosis of *A*. *baumannii* 1127417 significantly more than an isotype control antibody (*P* < 0.0001, Mann-Whitney *U* test) (Figure 3, B and C). In vivo protection was assayed using our standard murine bloodstream infection model with C3HeB/Fe mice (6, 19–22, 25–27). Mice (*n* = 8) were infected with *A*. *baumannii* 1127417 intravenously, then treated with either isotype control antibody or 1, 5, 50, or 150 μg MAb10. Most animals (6 of 8) given the isotype control died within 24 hours of infection. A single mouse died in the 1 μg MAb10 treatment group, but all other MAb10 treatment groups had no deaths (*P* < 0.01, log-rank test) (Figure 3D).

We further evaluated the efficacy of MAb10 using a Sprague-Dawley rat soft tissue wound model of infection (28–30). Rats (*n* = 4) were delivered 0, 5, or 10 mg/kg MAb10 intraperitoneally following subcutaneous infection with *A*. *baumannii* 1127417, and abscess fluid was sampled daily for 3 days. MAb10-treated groups had significantly reduced CFU/mL abscess fluid at all time points (*P* = 0.0286, Mann-Whitney *U* test for 5 mg/kg group, *P* = 0.0348, 2-tailed *t* test for 10 mg/kg group) (Figure 3E).

Given the success of murine MAb10 therapy, we humanized the MAb and subsequently tested the efficacy of humanized MAb10 against *A*. *baumannii* 1127417 in flow cytometry (Figure 3F; gating strategy in Supplemental Figure 1) and in our murine intravenous infection model and found that the binding and protective efficacy were maintained (*P* = 0.0253, log-rank test) (Figure 3G). The binding activity of humanized MAb10 was further characterized by assaying 30 additional isolates via flow cytometry and comparing the binding profile to murine MAb10. The binding was comparable in almost all cases, with a complete loss of binding activity observed in only 1 of the 30 isolates (Supplemental Table 1).

### MAb binding as assessed by flow cytometry correlates with in vivo protection.

In addition to the new MAb10, we have previously demonstrated that other MAbs, MAbC8, MAb65, BsAb C73 (a bispecific MAb combining 8 and 65), and MAb5, are also effective during lethal infection in vivo against strains of *A*. *baumannii* to which they bind well by flow cytometry, with approximately 80% binding (80% of events brighter than the isotype control) (6, 19–22). Given the availability of multiple MAbs, enabling a very broad coverage of strains, we sought to determine if a strain binding cutoff could be identified by flow cytometry that would enable accurate prediction of in vivo efficacy. Such a cutoff is intended to be used as a “susceptibility” test equivalent, akin to antimicrobial agents, to help clinical investigators conduct clinical trials with MAbs and future clinical deployment of MAbs by clinicians treating patients at the bedside.

To characterize such a susceptibility breakpoint, we first grouped MAbs and strains that either did or did not previously demonstrate efficacy during in vivo testing in published studies (6, 19–21) to compare efficacy to the percentage binding those strains showed in an in vitro flow cytometry assay (Table 2). We found that MAb/strain combinations with or without efficacy had clear patterns in strain binding: those MAb/strain combinations with high binding (85% or above) showed full protection in vivo, while those combinations with low binding (0%–3%) had no protection.

**Figure 4:**
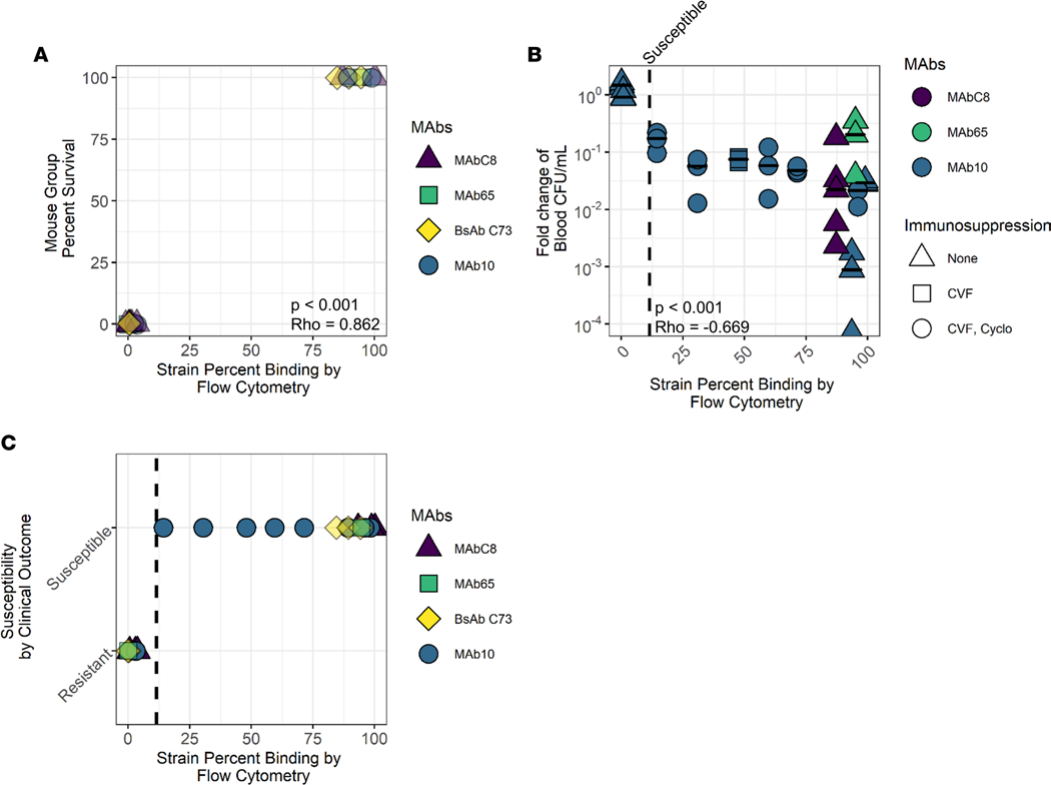
MAb binding of *A*. *baumannii* strains assessed by flow cytometry predicts in vivo treatment outcomes. (**A**) *Y* axis shows the percentage survival at 7 days after infection of mice when given MAb treatment, indicated by color. *X* axis shows the percentage binding (percentage of events in flow cytometry that were brighter than the isotype control). Each data point represents a group of *n* = 3 mice, with a total of 19 strain/MAb combinations being tested (listed in Table 2). *P* < 0.001, Rho = 0.862, 2-sided Spearman rank correlation test. (**B**) *Y* axis shows the fold-reduction of blood CFU, 2 hours after *A*. *baumannii* infection and treatment with 15 μg MAb. *X* axis shows percentage binding each strain with the MAb tested. Strain identities are indicated by color; immunosuppressive regimen used for each strain is shown by shape of the points. Bars indicate medians; each data point represents a single mouse. A total of 12 strain/MAb combinations are shown, using 11 different strains, *n* = 3–5 mice per strain/MAb combo. *P* < 0.001, Rho = –0.669, 2-sided Spearman rank correlation test. (**C**) *X* axis shows the percentage binding by flow cytometry. *Y* axis combines the data from 12 CFU experiments and 19 survival experiments to indicate strain susceptibility to MAb therapy. CVF, cobra venom factor; Cyclo, cyclophosphamide.

However, the *A*. *baumannii* strain/MAb combinations tested in in vivo survival studies were all clustered at very high (minimum of 85%) or very low (maximum of 3%) in vitro binding by flow cytometry, with no combinations tested that had intermediate binding (Figure 4A). On evaluation, this clustering effect was caused by the fact that very few global bacterial strains that were capable of causing lethal bloodstream infection in immune-competent mice had intermediate binding to any MAb. Since the intermediate-binding strains did not cause lethal infection, we conducted additional in vivo efficacy experiments in which the primary outcome was reduction of bacterial burden at 2 hours postinfection to enable additional comparisons of intermediate strain binding to efficacy.

MAb/strain combinations correlated very strongly with in vivo efficacy when tested for 7-day survival (*P* < 0.001, Spearman rank correlation test) or CFU reduction (*P* < 0.001) as the efficacy endpoint. We have previously established that an 0.5 log reduction of bacterial burden at 2 hours after infection resulted in 100% survival in 7-day survival studies (6). All MAbs that achieved at least a 0.5 log reduction in CFU/mL of blood had at least 14% binding to the target strain by flow cytometry (Figure 4B). The highest flow cytometry binding MAb/strain combination to exhibit no protection in a survival study was 3% (Figure 4A), indicating that the susceptibility breakpoint for flow cytometry binding lies between 3% and 14%. However, we found no combination of MAb/strain that had flow cytometry binding in that range, so we conservatively established the flow binding breakpoint definition of susceptibility at 14% based on both survival and blood CFU susceptibility data (Figure 4C). A MAb/strain binding cutoff of 14% or higher was both 100% sensitive and specific for predicting efficacy in vivo, resulting in an infinite positive likelihood ratio and negative likelihood ratio of 0.

These results suggested that 14% binding was a potentially highly accurate breakpoint for determining the susceptibility of a strain to MAb therapy, extrapolating from standard Clinical Laboratory and Standards Institute antimicrobial susceptibility breakpoint definitions.

### Rapid flow cytometry binding assay correlates with in vivo protection.

Having established reasonable susceptibility breakpoints that predicted accuracy, we sought to streamline the flow assay to make it more facile so that it could be rapidly deployed in a clinical laboratory setting. We specifically sought to reduce the assay to a single incubation step that could be carried out in a high-throughput manner by a laboratory technician working in a clinical microbiology laboratory.

**Figure 5:**
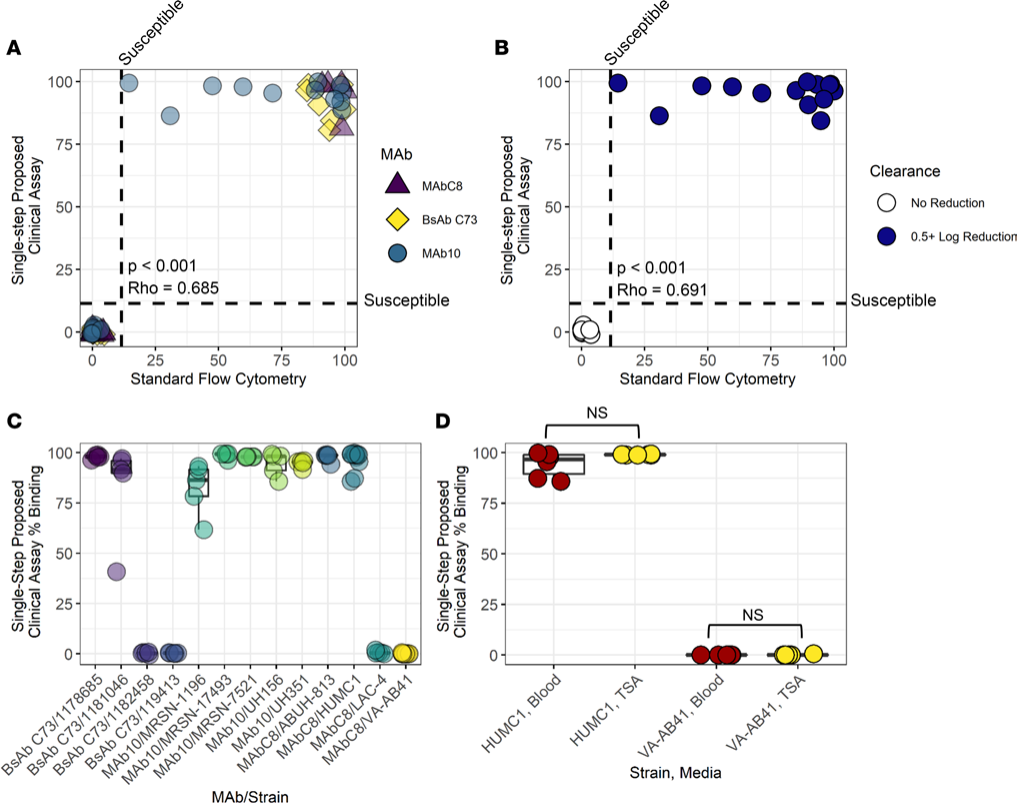
A rapid colony suspension flow cytometry assay produces consistent results that are similar to that of a standard flow assay. (**A**) Various strains (*n* = 60) of *A*. *baumannii* were assayed for binding with 3 different MAbs via the standard and rapid assay methods and show similar results with both assays. *P* < 0.001, Rho = 0.685, 2-sided Spearman rank correlation test. (**B**) *A*. *baumannii* strains (*n* = 24) that were assayed for in vivo susceptibility to our MAbs were tested via both standard and rapid flow assay methods. Coloration indicates susceptibility. *P* < 0.001, Rho = 0.691, 2-sided Spearman rank correlation test. (**C**) Consistency analysis of single-step flow cytometry. MAb/strain combinations (*n* = 13) were assayed 5 times from 5 separate colonies. Kappa = 0.943. Full statistical analyses available in Supplemental Table 2. (**D**) The single-step assay was done on *A*. *baumannii* strains isolated from either TSA or blood agar. No difference was observed between individual strains on either medium, *P* = 0.3095 and *P* = 0.2121, respectively, 2-sided Mann-Whitney *U* test.

Having developed a single-step workflow for flow binding, we conducted the assay on 58 strains of *A*. *baumannii* that have known binding reactions with MAb10, MAb C8, and BsAb C73, then compared these binding reactions directly to the results of the more time-consuming, labor-intensive standard flow cytometry assay. Comparison of the binding outcomes of MAb/strain pairs assayed by the standard, original method versus the streamlined, single-step method demonstrated very high correlations (Figure 5A, *P* < 0.001), though some strains did have higher binding in the single-step assay compared with the standard method. However, none of the changes in binding in the streamlined, single-step method altered the interpretation/prediction of in vivo efficacy as defined by reduction in blood bacteria burden, and none moved any MAb/strain combination from resistant to susceptible or vice versa using the 14% flow binding breakpoint (Figure 5B).

Given that the single-step method does not take into account the concentration of bacteria being assayed, we sought to evaluate the interassay variability of this method. Thirteen different MAb/strain combinations were assayed 5 times each via the single-step method, using separate isolated colonies for each assay. We found a high degree of consistency between assay results (Figure 5C and Supplemental Table 1). An overall Fleiss Kappa score of 0.943 was calculated, indicating near-perfect agreement between each assay’s prediction of susceptible/resistant outcomes.

Because it is standard practice for clinical laboratories to isolate bacterial colonies on blood agar, we sought to evaluate whether colonies isolated from this medium would produce the same result as those taken from the tryptic soy agar (TSA) plates we had been using. Colonies from 2 strains (HUMC1 and VA-AB41) were collected from TSA or blood agar and assayed via the single-step method, and no difference in output was detected between colonies from either medium for each strain (*P* = 0.3095 and *P* = 0.2121, respectively) (Figure 5D).

Given the high percentage binding of most strains assayed via the single-step method, we sought to determine if titrating the amount of MAb used in the procedure would produce binding that more closely mimicked the results of the previous flow cytometry method. We found that reducing the MAb concentration in this assay had inconsistent effects on different strains — likely due to different strains producing different sized colonies with different amounts of bacteria and extracellular products — and settled on maintaining the 10 μg/mL concentration, which produced the most consistent results (Supplemental Table 3).

### Latex bead agglutination assay provides rapid assessment of A. baumannii sensitivity to MAbs.

A single-step flow cytometry susceptibility test is possible to deploy in a clinical laboratory setting if a cytometer is available for the test. Given the typical location of flow cytometers in the hematology section of a clinical laboratory, and the possible inability to use infected materials in such cytometers, we also sought to develop a facile, high-throughput assay that did not depend on the availability of a flow cytometer. We therefore decided to develop a simple visual agglutination assay.

**Figure 6:**
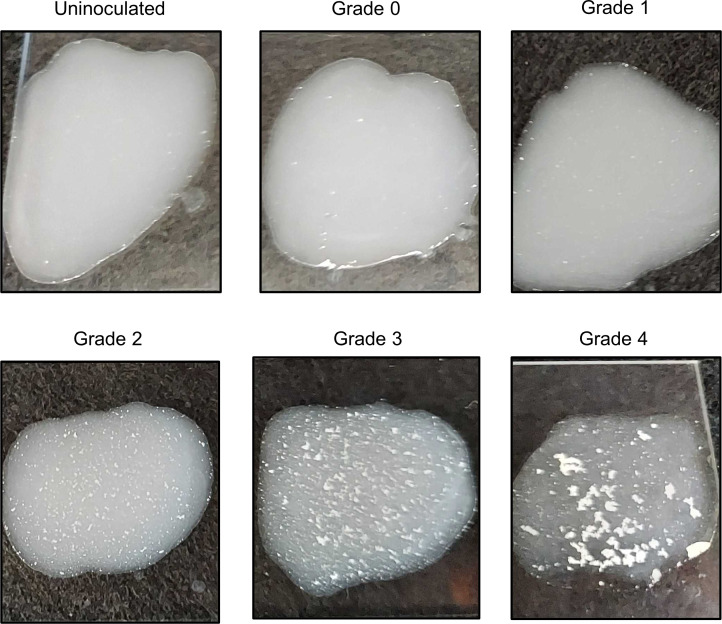
Depiction of the 5-point agglutination scale. Uninoculated: no bacteria was added, but the beads were still mixed with an inoculation loop for 10 seconds. Grade 0: no agglutination. The suspension remained white and translucent. Grade 1: slight agglutination. The suspension was translucent white but with very fine white grains emerging. Grade 2: significant agglutination. Most of the suspension was still clear-white, but moderate sized white grains appeared. Grade 3: high agglutination. The coloration of the fluid was reduced, as the latex was distributed into small white clumps. Grade 4: complete agglutination. The latex was largely distributed into large white clumps, leaving the remaining fluid mostly colorless.

**Figure 7:**
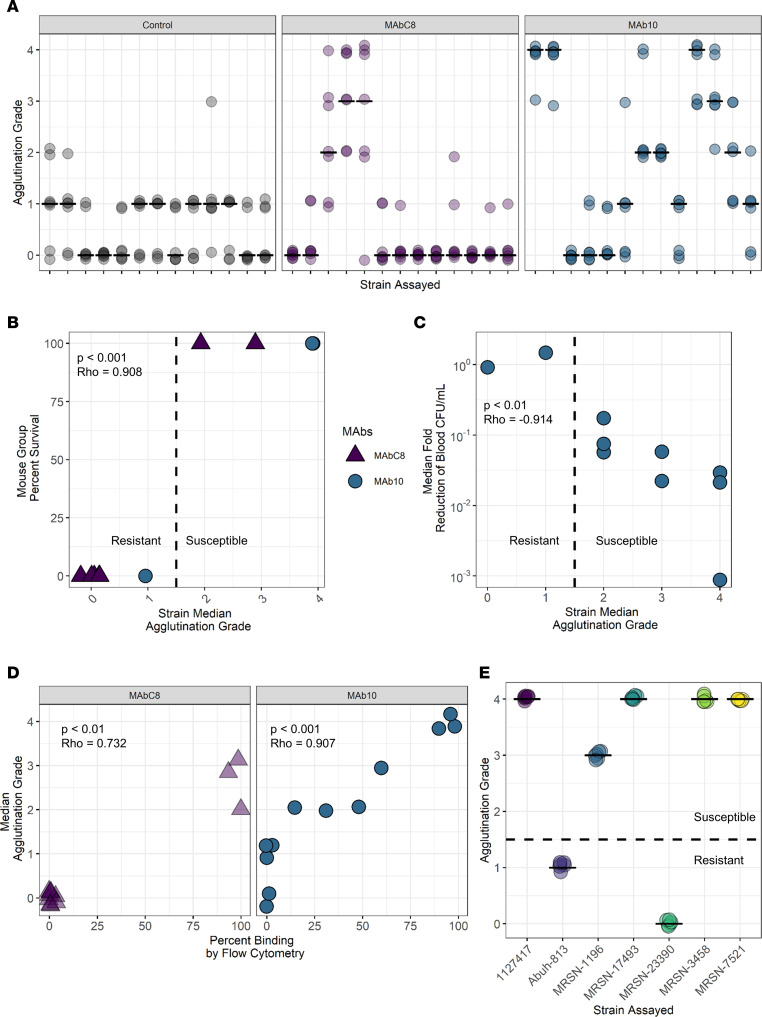
Latex bead agglutination assay corresponds with flow cytometry results and is predictive of in vivo success. Latex beads conjugated to MAb10 or MAbC8 were mixed with fresh colonies of *A*. *baumannii* in an agglutination assay that rapidly predicts MAb binding. (**A**) A total of 7 independent raters observed the agglutination reactions between 13 separate *A*. *baumannii* strains and 3 different MAb-conjugated bead types. *X* axis represents strains. *Y* axis shows the agglutination scores assigned by each rater. Bars display the median. Kappa inter-rater score = 0.784. (**B**) *Y* axis shows the percentage survival of mice treated with either MAb10 or MAbC8 following intravenous challenge with 1 of various *A*. *baumannii* strains (*n* = 9). *X* axis shows the agglutination reaction of these strains with beads conjugated with the MAb used in treatment. *P* < 0.001, Rho = 0.908, Spearman’s rank correlation coefficient. (**C**) *Y* axis shows the fold-reduction in blood CFU at 2 hours following intravenous *A*. *baumannii* infection followed by treatment with 15 μg MAb10. *X* axis shows the agglutination of these strains (*n* = 9) with MAb10 beads. *P* < 0.01, Rho = –0.914, 2-sided Spearman’s rank correlation coefficient. (**D**) Relating the percentage binding of strains (*n* = 13) in flow cytometry with the agglutination reaction of those same strains for either MAbC8 or MAb10. *P* < 0.01, Rho = 0.732 for C8 beads; *P* < 0.001, Rho = 0.907 for MAb10 beads, Spearman’s rank correlation coefficient. (**E**) Seven strains were used in repeat agglutination assays to test for interassay consistency. Six colonies per strain were separately assayed for agglutination with MAb10-conjugated beads and scored for their agglutination grade. Kappa interassay score = 1.

Latex beads were conjugated to MAbC8, MAb10, or isotype control antibodies, and a 5-point agglutination scale was developed (Figure 6) to quantify the observed agglutination, with a score of 0 indicating no agglutination whatsoever, and 4 indicating extensive agglutination of the beads in the presence of bacteria. Because reading agglutination assays can be subjective, 7 independent raters observed the agglutination reaction between all 3 prepared beads and 13 separate *A*. *baumannii* strains with known binding and in vivo characteristics (Figure 7A). The resultant data had a Fleiss Kappa score of 0.784, suggesting highly substantial inter-rater agreement. The median agglutination observations for each MAb/strain combination correlated strongly with both survival and CFU efficacy data (*P* < 0.001, *P* < 0.01, respectively) (Figure 7, B and C).

Furthermore, a clear line between protective and nonprotective combinations emerged between the agglutination scores of 1 and 2 (Figure 7, B and C). This finding was further supported by the strong correlation between median agglutination scores for both MAbC8 and MAb10 beads and flow cytometry binding data (Figure 7D), with the 14% binding susceptibility breakpoint coinciding with the agglutination score of 2. Hence, the agglutination assay can be interpreted as a binary, nominal readout of resistant (scores 0–1) or susceptible (scores 2–4). The sensitivity and specificity of a binary resistant/susceptible interpretation of the agglutination assay using grade 2 agglutination as the cutoff were 91.43% and 91.63%, respectively, resulting in a positive likelihood ratio of 25.6 and a negative likelihood ratio of 0.089.

To evaluate reproducibility of the assay, 6 colonies from 7 strains were assayed for agglutination with MAb10-conjugated beads and scored on the 0–4 graded scale as well as the binary resistant/susceptible interpretation (Figure 7E). All colonies exhibited the same agglutination pattern and sensitivity interpretation as other colonies from the same strain, with a Fleiss Kappa score of 1, indicating complete reproducibility between assays.

## Discussion

These 2 assays show promise for their ability to rapidly predict in vitro the success of a MAb therapy in an in vivo model of infection. Flow cytometry produces a sensitive and precise output, which extremely accurately predicts the success or failure of MAb therapy in survival studies, using strains with binding above or below the susceptibility breakpoint, respectively. The resultant data suggest that the susceptibility breakpoint for efficacy is between 3% and 14% binding, with a clear correlation between binding percentage and bacterial clearance in CFU studies, or survival in survival studies.

A shortcoming of flow cytometry as a clinical assay is that it is traditionally done with multiple washing steps performed on a mature culture to remove cellular debris and additional later washing steps to remove any unbound primary or secondary antibodies. In addition, multiple incubation steps are typically done, totaling to an approximately 2.5-hour-long procedure for assays using few samples. Here we have demonstrated that sensitivity or specificity of the assay are retained when using a truncated procedure that has only 1 half-hour incubation and no washing steps, making this an attractive option for a clinical susceptibility assay. It is worth noting, however, that as the proposed method puts live bacteria into the flow cytometer, frequent routine cleaning of the unit is important, as well as the addition of a mild sterilant such as 0.05% sodium azide to the antibody suspension.

Even faster than our rapid flow cytometry method, however, is the agglutination assay. A droplet of MAb-conjugated beads mixed with a single bacterial colony on a microscope slide produces evidence of MAb binding within 10 seconds. However, this rapidity comes at the cost of subjectivity. While flow cytometry can differentiate between bacterial strains that bind to MAbs with any percentage binding, the agglutination assay requires human interpretation, which can be subject to error. This is mathematically represented by the Kappa scores, which were used to evaluate interassay and inter-rater reproducibility of these assays. The single-step flow cytometry assay had a near-perfect Kappa score of 0.943, indicating excellent interassay reproducibility. The agglutination method was tested for both interassay and inter-rater reproducibility, with the former having a perfect Kappa score of 1 when multiple strains of *A*. *baumannii* were tested for interassay reproducibility, and a lower, but still impressive, score of 0.784 for interpretation by multiple raters. However, the raters used for the agglutination assay had no prior training or introduction to the assay. Therefore, thorough training in performing and interpreting the agglutination results would likely benefit the assay’s clinical utility. Additionally, given that flow cytometers are not presently used in clinical microbiology, the agglutination assay described here may be an appropriate contemporary assay until such a time that flow cytometers become available to clinical microbiologists.

These assays can be readily adapted to other MAbs that bind the surface of microbial pathogens. Nevertheless, while the concept of both the single-step flow cytometry and agglutination assays is easily translated to different pathogens and MAbs, we do not expect that the precise breakpoints detailed here would be the same for all organisms and antibodies. Even using the same simple assays, due to the vastly different pathologic/virulence properties of different microbial pathogens, each new therapeutic MAb would need to be investigated for its own binding or agglutination susceptibility breakpoints against its target organism in an appropriate animal model. The fundamental finding is that surface binding can be both rapidly and facilely assessed in assays that can be adapted to clinical laboratory settings and also highly predictive of whether a given MAb will effectively treat an infecting strain in vivo. These assays may therefore be useful to help discovery, development, and clinical deployment of MAbs for a variety of microbial pathogens in the future.

## Methods

### Generation of new MAb, MAb10.

We immunized BALB/c mice with a sublethal inoculum (2 × 10^5^ CFU/mouse) of a mixture of 30 clinical isolates of *A*. *baumannii* derived from different hospitals in the United States, Latin America, Asia, Europe, Australia, and New Zealand. These strains were selected because they were not bound by our prior MAbs C8 and C73. Hybridomas were generated and selected as we previously described (6, 19–22). Media from hybridomas were then assayed via flow cytometry for binding with the strains initially used in immunization, with the media taking the role of a primary antibody. After identifying hybridomas that produced promising MAbs, the MAb was purified by protein G affinity chromatography and assayed for binding against our full strain library. The resulting MAb, MAb10, was subsequently humanized by Absolute Antibody as we have previously described (22). In brief, variable regions were identified by reverse transcription PCR, using the Novagen mouse Ig degenerate primer set (catalog TB326) per the manufacturer’s instructions. Variable regions were aligned with the murine germline sequences from which they were derived and their human analogs. Humanized variable regions were grafted in silico to human IgG1 heavy- and light-chain constant regions and synthesized in mammalian expression vectors at GenScript.

### Bacterial inoculum preparation.

Bacteria were cultured overnight in tryptic soy broth (TSB) at 37°C, with shaking at 200 rpm. Subcultures were passaged at a 1:100 dilution and cultured for 3 hours to log phase under the same conditions, then washed 3 times with PBS and adjusted to the appropriate concentration in phosphate-buffered saline (PBS) prior to infection.

### Flow cytometry — standard method.

*A*. *baumannii* isolates were cultured overnight in TSB, and then a 1:100 dilution subculture was incubated in TSB for 3 hours. The cultures were then centrifuged at 4,000*g* for 5 minutes and washed 3 times in 10 mL PBS. Washed bacterial suspensions were then adjusted to OD600 0.5, then incubated with 1 μg/mL MAb or IgG control antibody (Thermo Fisher Scientific, catalog MAB002, clone 11711) for 30 minutes at 37°C. Following incubation, samples were centrifuged at 4,000*g* for 5 minutes and washed twice in an equal volume of PBS. Samples were then treated with 2 μg/mL secondary antibody with Alexa Fluor 647 (Thermo Fisher Scientific catalog A21235 when assaying murine MAbs, Thermo Fisher Scientific catalog A21445 for humanized MAbs) and incubated for an additional 30 minutes. Samples were then washed 2 times again as described above, and flow cytometry was performed using an Accuri C6 Plus (BD).

### Flow cytometry — rapid method.

Bacteria were streaked on TSA and incubated overnight at 37°C. Colonies were selected and suspended in 1 mL PBS with 0.05% sodium azide. MAbs or IgG control antibodies (Thermo Fisher Scientific, catalog MAB002, clone 11711) were added to a final concentration of 10 μg/mL, and Alexa Fluor 647 fluorescent IgG secondary antibodies (Thermo Fisher Scientific catalog A21235 when assaying murine MAbs, Thermo Fisher Scientific catalog A21445 for humanized MAbs) were added to a final concentration of 2 μg/mL. Samples were incubated at 37°C for 30 minutes. No wash steps were performed. Flow cytometry analysis was performed using an Accuri C6 Plus.

### Calculation of percentage binding.

Flow cytometry was performed as described above (either the standard or rapid method), using either an IgG isotype control antibody (Thermo Fisher Scientific, catalog MAB002, clone 11711), or one of our MAbs (MAbC8, MAb65, BsAb C73, or MAb10), as well as a secondary antibody conjugated to Alexa Fluor 647. The brightness of Alexa Fluor 647 was detected on the flow cytometer’s APC channel and interpreted in FlowJo software. Events were gated for brightness, with the low end of the gate set to include the brightest 1% of events in the IgG isotype control antibody–treated group and the high end of the gate set to the maximum brightness possible to detect. Percentage binding was calculated as the percentage of events in the MAb-treated group that fall within that gate and are therefore brighter than the control group. Each strain assayed was gated individually to account for slight variation in background fluorescence between strains. The method of calculating percentage binding was identical for both the standard and rapid flow cytometry methods. Supplemental Figure 1 provides a clear example of the gating strategy and calculation of percentage binding.

### Agglutination bead conjugation.

A 2.5 mL suspension of 1 μm carboxyl latex beads (Invitrogen catalog C37274), at a concentration of 4% solids (weight/volume), was washed 3 times via centrifugation at 4,000*g* for 20 minutes, resuspended in 0.5 M 4-(N-morpholino) ethanesulfonic acid buffer (MES) at pH 6.0 (Thermo Fisher Scientific catalog J62574.AK) between spins, followed by a final resuspension in 5 mL MES. A total of 5 mL purified MAb or control IgG antibody (Thermo Fisher Scientific, catalog MAB002, clone 11711) (1 mg/mL) was added, along with 500 mg of 1-ethyl-3-(3-dimethylaminopropyl) carbodiimide hydrochloride in a final volume of 10 mL MES. The mixture was incubated for 4 hours at room temperature with gentle rotation. The beads were then washed 3 times via centrifugation as above, followed by resuspension in 10 mL PBS, resulting in a final concentration of 1% latex solids. A working suspension of 0.5% latex solids (approximately 4.5 × 10^8^ beads/mL) was prepared by diluting 1:1 in PBS and used for downstream assays.

### Agglutination assay.

Bacteria were streaked on TSA and incubated overnight at 37°C. Colonies were selected and added into a 10 μL droplet of MAb-conjugated latex beads (4.5 × 10^8^ beads/mL) on a glass slide. The colonies were mixed with an inoculating loop (VWR, catalog 82051-146) for 10 seconds, and the agglutination result was graded as follows: Grade 0: no agglutination whatsoever; the suspension remained white and translucent. Grade 1: slight agglutination; the suspension was translucent white but with very fine white grains emerging. Grade 2: significant agglutination; most of the suspension was still clear-white, but moderate-sized white grains appeared. Grade 3: high agglutination; the coloration of the fluid is reduced, as the latex is distributed into small white clumps. Grade 4: complete agglutination; the coloration of the fluid is fully distributed into large white clumps, leaving the remaining fluid almost fully colorless. The agglutination assays photographed in Figure 6 were done using 100 μL droplets instead of 10 μL to aid visibility.

### Mouse intravenous infection model.

C3HeB/Fe mice were purchased from The Jackson Laboratory (strain 000658) and used for survival studies. Mice were between 9 and 12 weeks of age at the time of infection and weighed approximately 30 g. Mice were infected intravenously via the tail vein, followed by immediate administration of MAb or control treatment via the tail vein, and survival time was monitored for 7 days postinfection. Given variations in the virulence of *A*. *baumannii* strains, some studies utilized immunosuppression with either 0.5 mg/kg CVF or a combination of CVF and 230 mg/kg cyclophosphamide, delivered intraperitoneally 48 hours prior to infection (Supplemental Figure 2). In studies with low-virulence strains, all mice of all experimental groups were given identical immunosuppression.

### Rat subcutaneous abscess infection model.

Sprague-Dawley rats (Charles River Laboratories) were anesthetized and injected with 40 mL air to form a subcutaneous space, followed by injection of 1 mL 1% croton oil in a filter-sterilized vegetable oil vehicle. Over 10 days, this space formed an encapsulated, fluid-filled “pouch” with a volume of 8–12 mL. *A*. *baumannii* 1127417 (2.5 × 10^9^ CFU/mouse) was injected into the abscess, and mice were treated intraperitoneally with 5 or 10 mg/kg MAb10 or vehicle immediately following infection. Fluid aliquots of 0.5 mL were collected at time 0, as well as at 24-hour intervals, and assayed for CFU/mL by quantitative plating.

### Macrophage opsonophagocytosis assay.

RAW 264.7 murine macrophages (5 × 10^5^/well; ATCC) were cultured on a glass microscope slide coverslip and stimulated with 100 U/mL IFN-γ overnight in a humidified incubator at 37°C supplemented with 5% CO_2_. *A*. *baumannii* overnight cultures were subcultured to log phase, washed in PBS, and resuspended in Hanks’ balanced salt solution (HBSS) to 2 × 10^8^ CFU/mL. Cells were rinsed 3 times with HBSS, and bacteria were added to wells at a ratio of 20:1 (bacteria to macrophages) with 10% CD-1 (IMSCD1-COMPL; Innovative Research Inc.) mouse serum and 10 μg/mL MAb10 or control antibody (Thermo Fisher Scientific, catalog MAB002, clone 11711). Macrophages were washed 3 times with HBSS, fixed with 100% methanol, and Hema-3 stained according to the manufacturer’s protocol (Thermo Fisher Scientific). To quantitate bacteria per macrophage, coverslips were imaged on a Leica DMLS clinical microscope with a Leica ICC50 HD digital camera.

### Statistics.

Mouse survival curves were compared using the log-rank test (α = 0.05). Bacterial CFU in blood were compared with the Mann-Whitney test (α = 0.05). Correlations between strain binding of individual MAbs and MAb efficacy were run by Spearman rank correlation test (α = 0.05). Sensitivity of specific binding cutoff at predicting efficacy was defined as the number of MAb/strain combinations with a percentage of binding above the specified cutoff that showed protection in vivo divided by the total number of MAb/strain combinations with binding above the cutoff. Specificity was defined as the number of MAb/strain combinations with a percentage binding below the specified cutoff that showed no protection divided by the total number of MAb/strain combinations with binding below the cutoff. Positive likelihood ratio was defined as sensitivity/(1 – specificity), and negative likelihood ratio was defined as (1 – sensitivity)/specificity. Interrater reliability was calculated as Kappa scores, according to the Fleiss method (31).

### Study approval.

All mouse work was conducted following approval by the Institutional Animal Care and Use Committee at the University of Southern California, in compliance with the recommendations in the *Guide for the Care and Use of Laboratory Animals* of the NIH (revised 1985). All rat work was reviewed and approved by the Veterans Administration Institutional Animal Care Committee and the University at Buffalo-SUNY and was carried out in strict accordance with the recommendations in the guidelines delineated in the NIH *Guide for the Care and Use of Laboratory Animals* (revised 1985) and the “Ethics of Animal Experimentation Statement” (Canadian Council on Animal Care, July 1980) as monitored by the Institutional Animal Care and Use Committee. All efforts were made to minimize suffering. Veterinary care for the animals was supplied by the staff of the Veterans Administration Animal Facility under the direction of a fully licensed veterinarian.

### Data availability.

All data values included in all figures shown are fully available in Excel format in the Supporting Data Values spreadsheet included in this publication. All *A*. *baumannii* strains used, and their accession numbers where available, are listed in Supplemental Table 4. The Kaptive software used to analyze genome sequences is freely available on GitHub (https://github.com/klebgenomics/Kaptive; commit ID 563bfc4) by authors Kelly Wyres et al.

## Author contributions

MJS was responsible for designing and conducting experiments, acquiring and analyzing data, and writing the manuscript. NB was responsible for conducting experiments and acquiring data. EB was responsible for conducting experiments. JY was responsible for designing experiments. UCM and TAR were responsible for designing and conducting experiments and analyzing data. BML was responsible for designing experiments and analyzing data. BS was responsible for designing experiments, analyzing data, and writing the manuscript.

## Supplementary Material

Supplemental data

Supporting data values

## Figures and Tables

**Table 1:**
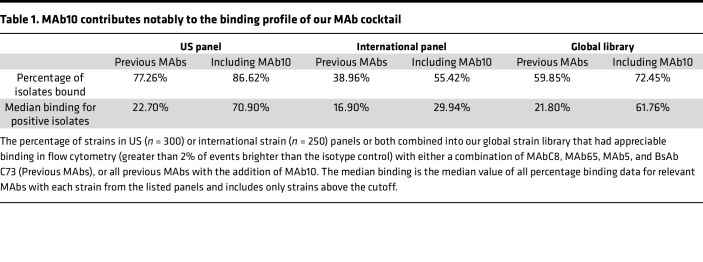
MAb10 contributes notably to the binding profile of our MAb cocktail

**Table 2:**
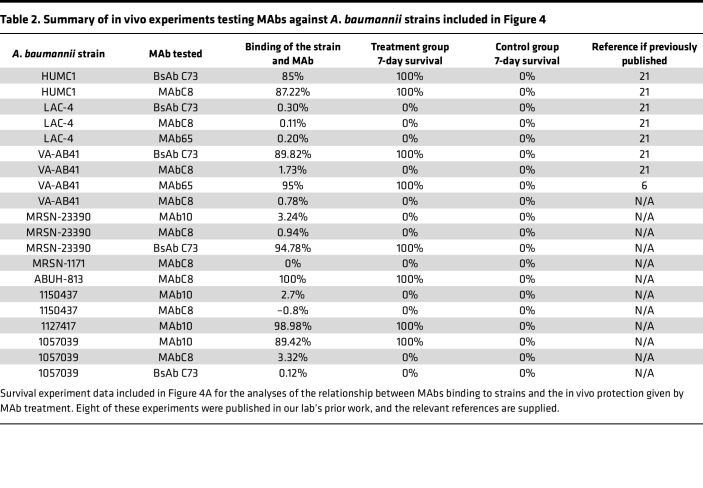
Summary of in vivo experiments testing MAbs against *A*. *baumannii* strains included in Figure 4

## Version 1 01/23/2024

Electronic publication
