# Supplementary material for: Clinical assays rapidly predict bacterial susceptibility to monoclonal antibody therapy
Source: JCI Insight. 2024 Jan 23;9(2):e174799. doi: 10.1172/jci.insight.174799 (PMC10906227; doi:10.1172/jci.insight.174799)
Supplement: Supplemental data [file jciinsight-9-174799-s021.pdf]

Supplement

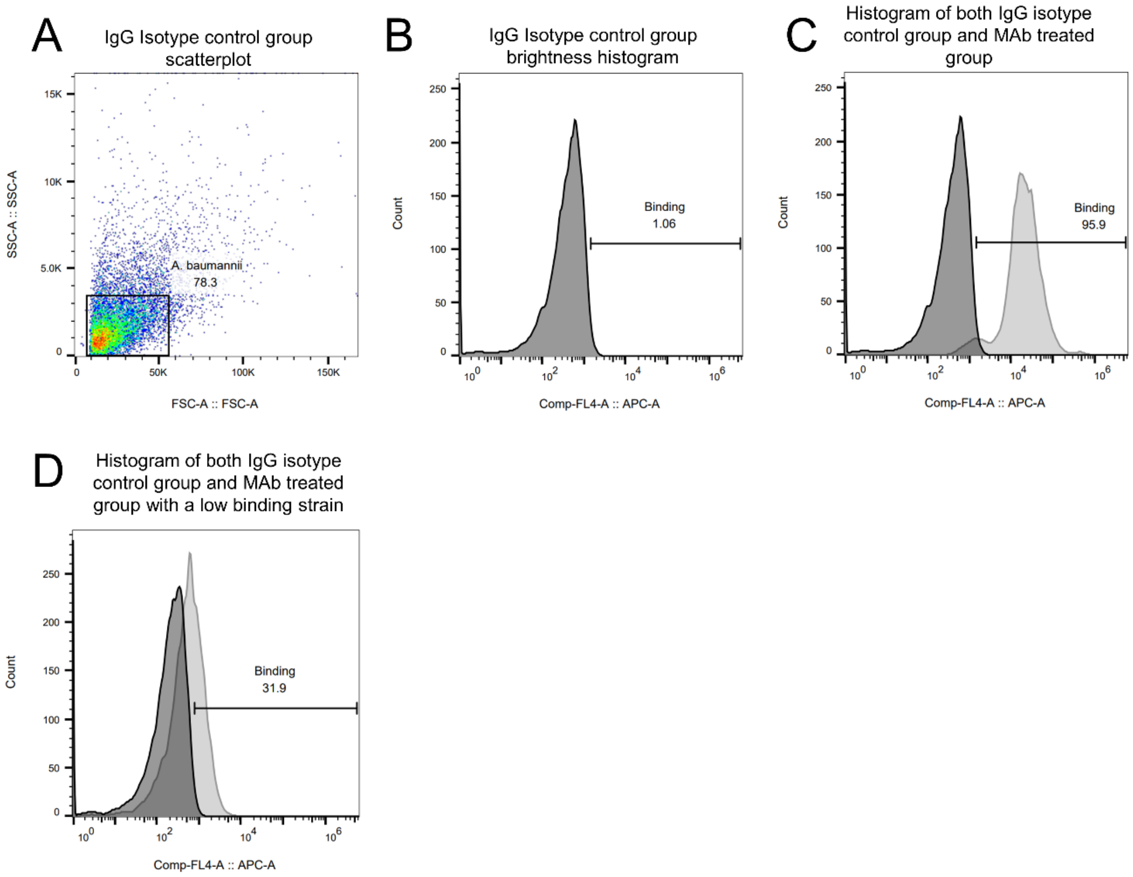

Figure S1. Bacterial flow cytometry gating strategy via Flowjo software and acquisition of percent binding. The flow cytometry events (10,000 events per sample) of the IgG isotype control group were first viewed on a forward scatter/side scatter plot, and a box gate was applied to select only the core events, eliminating any excessively large or granular events. This was done to prevent bacterial aggregates from contributing to the binding data (A). The events gated as in figure A were plotted as a histogram with the X axis indicating brightness on the flow cytometer's APC channel. A gate was produced to include all events brighter than the control group, as well as the brightest 1% of events within the control group (B). These gates were then applied to the MAb treatment group, and the percentage of events within the fluorescence gate were interpreted as percent binding (C). This process yielded different but comparable results for each isolate, and results in a wide range of binding reactions, with lower binding strains having heavy overlap between the control and MAb treated histograms, resulting in a lower percent binding (D). Subtle variations in the background fluorescence of the control group between strains used in figures B/C and D necessitate normalizing the results using a control group for each strain individually.

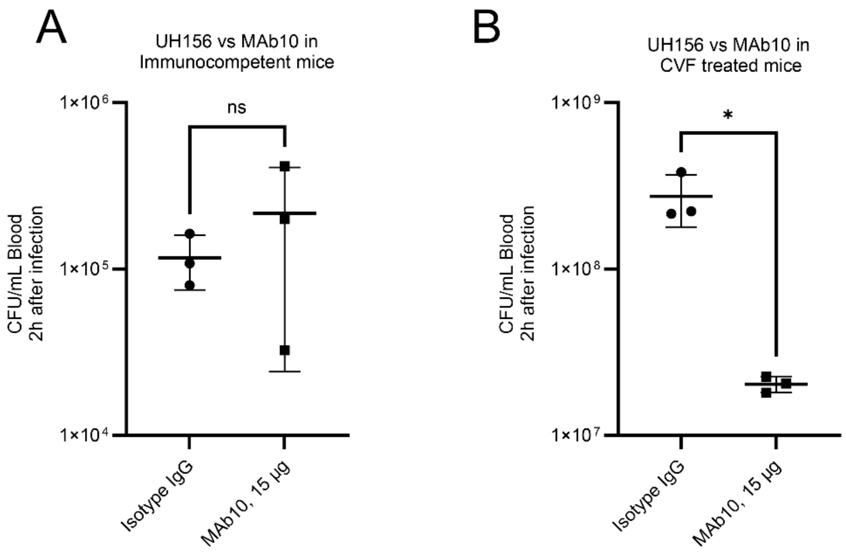

Figure S2. Explanation of Immunosuppressive regimen. Some *A. baumannii* strains were incapable of producing lethal infection, or even maintaining a significant enough presence in the bloodstream following infection to accurately assay the effects of MAb treatment. (A) Immunocompetent Mice (n=3/group) given 3.75e8 CFU of strain UH156 cleared the infection so efficiently that there was no difference in blood CFU between the control and MAb10 treated groups. However, mice given the same infective dose following cobra venom factor treatment to eliminate complement were unable to clear the infection with this same avirulent strain without MAb10 treatment. We do not anticipate that the immunosuppressive regimen has any impact on MAb efficacy beyond enabling these sublethal strains to be used in our infection model, given that we have previously established MAb efficacy in mice with both complement and neutrophil depletions (19-21).

A. baumannii Isolate	Murine MAb10 percent binding	Humanized MAb10 percent binding	Murine-Human percent binding differential
1127331	98.4	41.1	57.3
1127417	100	100	0
1157987	97.53	97.54	-0.01
1180986	98.02	86.02	12
AR286	97.9	97.37	0.53
AR291	98.16	98.17	-0.01
AR298	97.98	96.3	1.68
AR301	97.83	98.25	-0.42
AR303	97.44	95.96	1.48
AR308	97.57	96.28	1.29
MRSN-1196	30.84	0.64	30.2
MRSN-7521	52.2	55.87	-3.67
MRSN-7735	97.82	93.63	4.19
MRSN-17493	40.7	52.65	-11.95
MRSN-32797	97.34	95.52	1.82
UH086	98.31	97.25	1.06
UH116	98.46	97.03	1.43
UH125	97.86	91.67	6.19
UH136	97.9	92.52	5.38
UH203	98.44	96.65	1.79
UH206	98.38	97.77	0.61
UH210	98	96.36	1.64
UH213	98.58	97.97	0.61
UH218	98.58	94.92	3.66
VA-Ab13	97.62	97.63	-0.01
VA-Ab14	98.64	97.83	0.81
VA-Ab15	98.04	96.72	1.32
VA-Ab33	98.03	98.37	-0.34
VA-Ab78	98.99	98.2	0.79
VA-Ab85	98.81	94.8	4.01

Table S1. Flow cytometry comparison of humanized and murine MAb10. Percent binding of various *A. baumannii* isolates (n=30) with either humanized or murine MAb10 were compared. Assays were conducted identically. The fourth column shows the differential between murine and humanized MAb10 binding of each strain (calculated as murine MAb10 % binding – humanized MAb10 % binding). Strains with >10% difference between murine and humanized MAb10 are shown in grey.

MAb/Strain	Coefficient of Variation	Range	Variance
MAbC8/ABUH-813	1.86%	4.74	3.973069
MAbC8/HUMC1	4.80%	14.01	21.50595
MAbC8/LAC-4	88.74%	1.89	0.065319
MAbC8/VA-AB41	337.15%	0.6607	0.032597
BsAb C73/119413	51.23%	0.515	0.03328
BsAb C73/1081323	186.96%	19.65	71.47542
BsAb C73/1174866	106.98%	1.169	0.204268
BsAb C73/1178685	0.92%	2.6	1.005069
BsAb C73/1181046	25.61%	56.16	550.8906
BsAb C73/1182458	169.01%	1.44	0.219819
MAb10/MRSN-1196	13.97%	31.5	127.1275
MAb10/MRSN-17493	1.30%	3.2	1.92
MAb10/MRSN-7521	0.08%	0.2	0.0075
MAb10/UH156	5.61%	13.5	28.26188
MAb10/UH351	1.68%	4.4	3.1475

Table S2. Statistics of the consistency analysis of single-step flow cytometry as in Figure 5c. Coefficient of variation, variance, and range of all MAb/Strain combos assayed in the assessment of inter-assay consistency of the single-step flow cytometry assay are shown. Coefficient of variation is inflated by the low binding values of strains not bound, or weakly bound by our MAbs.

Strain	10 μg/mL MAb10	5 μg/mL MAb10	1 μg/mL MAb10	0 μg/mL MAb10
**MRSN-17493**	100	100	86.3	1.03
**MRSN-1196**	92.1	94.2	10.1	1.29
**MRSN-7521**	99.9	95.6	36.2	1.04
**UH351**	97.8	65.8	15.5	1.19
**UH156**	100	100	99	0.22

Table S3. MAb concentration titration analysis of single-step flow cytometry. Five *A. baumannii* strains were assayed for binding via the single-step flow cytometry method using 10, 5, 1 or 0 μg/mL MAb10, with a standard 2 μg/mL of secondary antibody. Titrating the MAb10 concentration had different effects on different strains, so the 10 μg/mL dose was used to maintain consistency.

Isolate	Panel	Origin	Accession Number	Use
747507	International	China	N/A	Binding analysis, Immunization
747510	International	China	N/A	Binding analysis
747592	International	China	N/A	Binding analysis
747796	International	China	N/A	Binding analysis
747912	International	China	N/A	Binding analysis, Immunization
747913	International	China	N/A	Binding analysis, Immunization
747926	International	China	N/A	Binding analysis, Immunization
748337	International	China	N/A	Binding analysis, Immunization
748366	International	China	N/A	Binding analysis
815013	International	China	N/A	Binding analysis
815071	International	China	N/A	Binding analysis
815110	International	China	N/A	Binding analysis
815258	International	China	N/A	Binding analysis
815262	International	China	N/A	Binding analysis
815297	International	China	N/A	Binding analysis
815513	International	China	N/A	Binding analysis
815637	International	China	N/A	Binding analysis
815702	International	China	N/A	Binding analysis
815732	International	China	N/A	Binding analysis
815782	International	China	N/A	Binding analysis
815992	International	China	N/A	Binding analysis
816012	International	China	N/A	Binding analysis
816081	International	China	N/A	Binding analysis
816289	International	China	N/A	Binding analysis
816351	International	China	N/A	Binding analysis
816376	International	China	N/A	Binding analysis
817106	International	China	N/A	Binding analysis
817238	International	China	N/A	Binding analysis
817258	International	China	N/A	Binding analysis
817382	International	China	N/A	Binding analysis
817475	International	China	N/A	Binding analysis
817718	International	China	N/A	Binding analysis
817774	International	China	N/A	Binding analysis, Immunization
1046098	International	Germany	N/A	Binding analysis, Immunization
1049062	International	Slovenia	N/A	Binding analysis
1049478	International	Slovenia	N/A	Binding analysis
1050572	International	Malaysia	N/A	Binding analysis
1051472	International	Italy	N/A	Binding analysis
1051765	International	Hungary	N/A	Binding analysis, Immunization
1051970	International	Hungary	N/A	Binding analysis

1052043	International	Germany	N/A	Binding analysis
1053486	International	Portugal	N/A	Binding analysis
1053664	International	Portugal	N/A	Binding analysis
1054973	International	Philippines	N/A	Binding analysis
1057010	International	Argentina	N/A	Binding analysis, Immunization
1057039	International	Argentina	N/A	Binding analysis, *in vivo* tests, Binding analysis, Immunization
1058484	International	Italy	N/A	Binding analysis
1059007	International	Spain	N/A	Binding analysis
1059884	International	UK	N/A	Binding analysis
1060672	International	Italy	N/A	Binding analysis
1064048	International	Australia	N/A	Binding analysis
1064859	International	Australia	N/A	Binding analysis
1066585	International	Sweden	N/A	Binding analysis
1067504	International	Australia	N/A	Binding analysis
1068987	International	UK	N/A	Binding analysis
1070754	International	Chile	N/A	Binding analysis, Immunization
1071498	International	Ireland	N/A	Binding analysis
1071861	International	Japan	N/A	Binding analysis
1071864	International	Japan	N/A	Binding analysis
1072139	International	Japan	N/A	Binding analysis
1072142	International	Japan	N/A	Binding analysis
1072821	International	Czech Republic	N/A	Binding analysis
1073791	International	UK	N/A	Binding analysis
1074199	International	Belgium	N/A	Binding analysis, Immunization
1074459	International	Korea	N/A	Binding analysis
1074634	International	Korea	N/A	Binding analysis, Immunization
1074724	International	Korea	N/A	Binding analysis
1074748	International	Korea	N/A	Binding analysis
1074887	International	Korea	N/A	Binding analysis
1075221	International	France	N/A	Binding analysis
1075260	International	France	N/A	Binding analysis
1076038	International	Brazil	N/A	Binding analysis
1076093	International	Brazil	N/A	Binding analysis
1076291	International	Brazil	N/A	Binding analysis
1076482	International	France	N/A	Binding analysis, Immunization
1076681	International	France	N/A	Binding analysis
1079113	International	Brazil	N/A	Binding analysis
1079126	International	Brazil	N/A	Binding analysis
1079607	International	Japan	N/A	Binding analysis
1079611	International	Japan	N/A	Binding analysis
1079767	International	Argentina	N/A	Binding analysis

1080109	International	Japan	N/A	Binding analysis
1080111	International	Japan	N/A	Binding analysis
1080230	International	Italy	N/A	Binding analysis
1080928	International	New Zealand	N/A	Binding analysis
1081274	International	Thailand	N/A	Binding analysis
1081275	International	Thailand	N/A	Binding analysis
1081316	International	Thailand	N/A	Binding analysis
1081317	International	Thailand	N/A	Binding analysis
1081318	International	Thailand	N/A	Binding analysis, Immunization
1081321	International	Thailand	N/A	Binding analysis
1081323	International	Thailand	N/A	Binding analysis
1081400	International	Thailand	N/A	Binding analysis, Immunization
1081856	International	Germany	N/A	Binding analysis, Immunization
1082029	International	Germany	N/A	Binding analysis
1082597	International	Germany	N/A	Binding analysis, Immunization
1082957	International	Greece	N/A	Binding analysis
1083037	International	Greece	N/A	Binding analysis
1083198	International	Taiwan	N/A	Binding analysis, Immunization
1083246	International	Taiwan	N/A	Binding analysis
1083383	International	Taiwan	N/A	Binding analysis
1084339	International	Russia	N/A	Binding analysis
1084397	International	Russia	N/A	Binding analysis
1084501	International	Russia	N/A	Binding analysis
1084583	International	Russia	N/A	Binding analysis
1084789	International	Belarus	N/A	Binding analysis
1085259	International	Romania	N/A	Binding analysis
1085280	International	Romania	N/A	Binding analysis
1086153	International	France	N/A	Binding analysis
1086362	International	France	N/A	Binding analysis
1086367	International	France	N/A	Binding analysis
1086858	International	Mexico	N/A	Binding analysis
1086903	International	Mexico	N/A	Binding analysis
1087377	International	Vietnam	N/A	Binding analysis
1087529	International	Vietnam	N/A	Binding analysis
1087564	International	Vietnam	N/A	Binding analysis
1087774	International	Panama	N/A	Binding analysis
1090896	International	Mexico	N/A	Binding analysis
1092847	International	Germany	N/A	Binding analysis
1096635	International	UK	N/A	Binding analysis
1096848	International	Portugal	N/A	Binding analysis
1098233	International	Australia	N/A	Binding analysis

1098733	International	Hungary	N/A	Binding analysis
1099017	International	UK	N/A	Binding analysis
1099731	International	Mexico	N/A	Binding analysis
1102304	International	France	N/A	Binding analysis
1102519	International	Malaysia	N/A	Binding analysis
1103665	International	Sweden	N/A	Binding analysis
1105437	International	Poland	N/A	Binding analysis
1109913	International	Spain	N/A	Binding analysis
1112707	International	Turkey	N/A	Binding analysis
1112954	International	Australia	N/A	Binding analysis
1114133	International	Germany	N/A	Binding analysis
1115614	International	New Zealand	N/A	Binding analysis
1115776	International	New Zealand	N/A	Binding analysis
1116232	International	Thailand	N/A	Binding analysis
1116239	International	Thailand	N/A	Binding analysis
1116527	International	Thailand	N/A	Binding analysis
1116531	International	Thailand	N/A	Binding analysis
1116836	International	Australia	N/A	Binding analysis
1118376	International	Australia	N/A	Binding analysis
1118686	International	Korea	N/A	Binding analysis
1119413	International	Ireland	N/A	Binding analysis
1119539	International	Ireland	N/A	Binding analysis
1121642	International	UK	N/A	Binding analysis
1122323	International	Argentina	N/A	Binding analysis
1124049	International	Philippines	N/A	Binding analysis
1124211	International	Philippines	N/A	Binding analysis
1124614	International	Russia	N/A	Binding analysis
1124675	International	Belarus	N/A	Binding analysis
1125119	International	Russia	N/A	Binding analysis
1125623	International	Taiwan	N/A	Binding analysis
1125675	International	Taiwan	N/A	Binding analysis, Immunization
1125683	International	Taiwan	N/A	Binding analysis, Immunization
1125896	International	Germany	N/A	Binding analysis
1126233	International	Vietnam	N/A	Binding analysis
1126239	International	Vietnam	N/A	Binding analysis
1126295	International	Vietnam	N/A	Binding analysis
1126344	International	Vietnam	N/A	Binding analysis
1126345	International	Vietnam	N/A	Binding analysis
1126369	International	Vietnam	N/A	Binding analysis
1126501	International	Vietnam	N/A	Binding analysis, Immunization
1126502	International	Vietnam	N/A	Binding analysis

1126509	International	Vietnam	N/A	Binding analysis
1126515	International	Vietnam	N/A	Binding analysis
1126516	International	Vietnam	N/A	Binding analysis
1126524	International	Vietnam	N/A	Binding analysis
1126615	International	Argentina	N/A	Binding analysis
1127331	International	Brazil	N/A	Binding analysis, Immunization
1127417	International	Brazil	N/A	Binding analysis, *in vivo* tests, Immunization
1127911	International	Brazil	N/A	Binding analysis
1128201	International	Greece	N/A	Binding analysis
1128390	International	Australia	N/A	Binding analysis
1128503	International	Australia	N/A	Binding analysis
1128704	International	France	N/A	Binding analysis
1131023	International	Italy	N/A	Binding analysis
1131223	International	Panama	N/A	Binding analysis
1131762	International	Germany	N/A	Binding analysis
1152947	International	Germany	N/A	Binding analysis
1154347	International	Sweden	N/A	Binding analysis
1156243	International	Hungary	N/A	Binding analysis
1156420	International	Hungary	N/A	Binding analysis
1156499	International	Australia	N/A	Binding analysis
1156553	International	Australia	N/A	Binding analysis
1156943	International	Mexico	N/A	Binding analysis
1157792	International	Mexico	N/A	Binding analysis
1157987	International	Italy	N/A	Binding analysis
1158457	International	Czech Republic	N/A	Binding analysis
1159347	International	UK	N/A	Binding analysis
1159494	International	Italy	N/A	Binding analysis
1160141	International	France	N/A	Binding analysis
1160980	International	Spain	N/A	Binding analysis
1162491	International	Poland	N/A	Binding analysis
1162714	International	Spain	N/A	Binding analysis
1162788	International	Spain	N/A	Binding analysis
1163235	International	Spain	N/A	Binding analysis
1165688	International	Portugal	N/A	Binding analysis
1167695	International	Belgium	N/A	Binding analysis
1171508	International	Malaysia	N/A	Binding analysis
1172743	International	Malaysia	N/A	Binding analysis
1172788	International	Thailand	N/A	Binding analysis
1172798	International	Thailand	N/A	Binding analysis
1173847	International	Thailand	N/A	Binding analysis
1174192	International	Philippines	N/A	Binding analysis

1174866	International	UK	N/A	Binding analysis
1174913	International	Greece	N/A	Binding analysis
1174945	International	Greece	N/A	Binding analysis
1176985	International	Greece	N/A	Binding analysis
1177238	International	Korea	N/A	Binding analysis
1177358	International	Korea	N/A	Binding analysis
1177473	International	Panama	N/A	Binding analysis
1177495	International	Panama	N/A	Binding analysis
1177592	International	Panama	N/A	Binding analysis
1178685	International	Panama	N/A	Binding analysis
1179185	International	Argentina	N/A	Binding analysis
1180013	International	Chile	N/A	Binding analysis
1180619	International	Greece	N/A	Binding analysis
1180949	International	Italy	N/A	Binding analysis
1180979	International	Taiwan	N/A	Binding analysis
1180981	International	Taiwan	N/A	Binding analysis, Immunization
1180984	International	Taiwan	N/A	Binding analysis
1180986	International	Taiwan	N/A	Binding analysis
1181046	International	Taiwan	N/A	Binding analysis
1181055	International	Taiwan	N/A	Binding analysis
1181082	International	Taiwan	N/A	Binding analysis
1181133	International	Taiwan	N/A	Binding analysis
1181291	International	Taiwan	N/A	Binding analysis
1181296	International	Portugal	N/A	Binding analysis
1181360	International	Portugal	N/A	Binding analysis
1181401	International	Chile	N/A	Binding analysis
1181422	International	Portugal	N/A	Binding analysis
1182458	International	Portugal	N/A	Binding analysis
1182471	International	Israel	N/A	Binding analysis
1182538	International	Israel	N/A	Binding analysis
1182550	International	Israel	N/A	Binding analysis
1183913	International	Israel	N/A	Binding analysis
1184244	International	Romania	N/A	Binding analysis
1185249	International	Germany	N/A	Binding analysis
1185250	International	Germany	N/A	Binding analysis
1185261	International	Germany	N/A	Binding analysis
1185713	International	Germany	N/A	Binding analysis
1188434	International	Brazil	N/A	Binding analysis
1188882	International	Ireland	N/A	Binding analysis
1190024	International	Turkey	N/A	Binding analysis
1190916	International	Germany	N/A	Binding analysis

1190918	International	Israel	N/A	Binding analysis
1190921	International	Israel	N/A	Binding analysis
1190923	International	Israel	N/A	Binding analysis
1190927	International	Israel	N/A	Binding analysis
3048882	USA	USA	N/A	Binding analysis
3048955	USA	USA	N/A	Binding analysis
3048981	USA	USA	N/A	Binding analysis
3049142	USA	USA	N/A	Binding analysis, Immunization
3049143	USA	USA	N/A	Binding analysis
3049297	USA	USA	N/A	Binding analysis
3049334	USA	USA	N/A	Binding analysis
3049353	USA	USA	N/A	Binding analysis
AB0057	USA	USA	N/A	Binding analysis
AB0061	USA	USA	N/A	Binding analysis
AB0068	USA	USA	N/A	Binding analysis
AB0071	USA	USA	N/A	Binding analysis
AB0072	USA	USA	N/A	Binding analysis
AB0074	USA	USA	N/A	Binding analysis
AB0093	USA	USA	N/A	Binding analysis
AB020	USA	USA	N/A	Binding analysis
AB044	USA	USA	N/A	Binding analysis
AB046	USA	USA	N/A	Binding analysis
AB074	USA	USA	N/A	Binding analysis
AB39	USA	USA	N/A	Binding analysis
AB5075	USA	USA	SAMN21212520	K-type binding analysis
AR273	USA	USA	SAMN04901663	K-type binding analysis
AR274	USA	USA	SAMN04901664	K-type binding analysis
AR275	USA	USA	SAMN04901665	K-type binding analysis
AR276	USA	USA	SAMN04901666	K-type binding analysis
AR277	USA	USA	SAMN04901667	K-type binding analysis
AR278	USA	USA	SAMN04901668	K-type binding analysis
AR279	USA	USA	SAMN04901669	K-type binding analysis
AR280	USA	USA	SAMN04901670	K-type binding analysis
AR281	USA	USA	SAMN04901671	K-type binding analysis
AR282	USA	USA	SAMN04901672	K-type binding analysis
AR283	USA	USA	SAMN04901673	K-type binding analysis
AR284	USA	USA	SAMN04901674	K-type binding analysis
AR285	USA	USA	SAMN04901675	K-type binding analysis
AR286	USA	USA	SAMN04901676	K-type binding analysis
AR287	USA	USA	SAMN04901677	K-type binding analysis
AR288	USA	USA	SAMN04901678	K-type binding analysis

AR289	USA	USA	SAMN04901679	K-type binding analysis
AR290	USA	USA	SAMN04901680	K-type binding analysis
AR291	USA	USA	SAMN04901681	K-type binding analysis
AR292	USA	USA	SAMN04901682	K-type binding analysis
AR293	USA	USA	SAMN04901683	K-type binding analysis
AR294	USA	USA	SAMN04901684	K-type binding analysis
AR295	USA	USA	SAMN04901685	K-type binding analysis
AR296	USA	USA	SAMN04901686	K-type binding analysis
AR297	USA	USA	SAMN04901687	K-type binding analysis
AR298	USA	USA	SAMN04901688	K-type binding analysis
AR299	USA	USA	SAMN04901689	K-type binding analysis
AR300	USA	USA	SAMN04901690	K-type binding analysis
AR301	USA	USA	SAMN04901691	K-type binding analysis
AR302	USA	USA	SAMN04901692	K-type binding analysis
AR303	USA	USA	SAMN04901693	K-type binding analysis
AR304	USA	USA	SAMN04901694	K-type binding analysis
AR305	USA	USA	SAMN04901695	K-type binding analysis
AR306	USA	USA	SAMN04901696	K-type binding analysis
AR307	USA	USA	SAMN04901697	K-type binding analysis
AR308	USA	USA	SAMN04901698	K-type binding analysis
AR309	USA	USA	SAMN04901699	K-type binding analysis
AR310	USA	USA	SAMN04901700	K-type binding analysis
AR311	USA	USA	SAMN04901701	K-type binding analysis
AR312	USA	USA	SAMN04901702	K-type binding analysis
AR313	USA	USA	SAMN04901703	K-type binding analysis
ARLG-1314	USA	USA	N/A	Binding analysis
ARLG-1774	USA	USA	N/A	Binding analysis, Immunization
ARLG-1775	USA	USA	N/A	Binding analysis
ARLG-1777	USA	USA	N/A	Binding analysis
ARLG-1783	USA	USA	N/A	Binding analysis
ARLG-1787	USA	USA	N/A	Binding analysis
ARLG-1788	USA	USA	N/A	Binding analysis
ARLG-1791	USA	USA	N/A	Binding analysis
ARLG-1801	USA	USA	N/A	Binding analysis
ARLG-1802	USA	USA	N/A	Binding analysis
ARLG-1808	USA	USA	N/A	Binding analysis
ARLG-1809	USA	USA	N/A	Binding analysis
ARLG-1811	USA	USA	N/A	Binding analysis
ARLG-1815	USA	USA	N/A	Binding analysis
ARLG-1817	USA	USA	N/A	Binding analysis
ARLG-1818	USA	USA	N/A	Binding analysis

ARLG-1819	USA	USA	N/A	Binding analysis
ARLG-1821	USA	USA	N/A	Binding analysis
ARLG-1837	USA	USA	N/A	Binding analysis
ARLG-1842	USA	USA	N/A	Binding analysis
ARLG-1844	USA	USA	N/A	Binding analysis
ARLG-1848	USA	USA	N/A	Binding analysis
ARLG-1850	USA	USA	N/A	Binding analysis
ARLG-1852	USA	USA	N/A	Binding analysis
ARLG-1854	USA	USA	N/A	Binding analysis
ARLG-1858	USA	USA	N/A	Binding analysis
ARLG-1860	USA	USA	N/A	Binding analysis
ARLG-1863	USA	USA	N/A	Binding analysis
ARLG-1866	USA	USA	N/A	Binding analysis
ARLG-1867	USA	USA	N/A	Binding analysis
ARLG-1868	USA	USA	N/A	Binding analysis
ARLG-1872	USA	USA	N/A	Binding analysis
ARLG-1876	USA	USA	N/A	Binding analysis
ARLG-1878	USA	USA	N/A	Binding analysis
ARLG-1883	USA	USA	N/A	Binding analysis
ARLG-1884	USA	USA	N/A	Binding analysis
ARLG-1885	USA	USA	N/A	Binding analysis
ARLG-1886	USA	USA	N/A	Binding analysis
ARLG-1887	USA	USA	N/A	Binding analysis
ARLG-1889	USA	USA	N/A	Binding analysis
ARLG-1890	USA	USA	N/A	Binding analysis
ARLG-1893	USA	USA	N/A	Binding analysis
ARLG-1894	USA	USA	N/A	Binding analysis
ARLG-1897	USA	USA	N/A	Binding analysis
ARLG-1903	USA	USA	N/A	Binding analysis
ARLG-1904	USA	USA	N/A	Binding analysis
ARLG-1908	USA	USA	N/A	Binding analysis
ARLG-1911	USA	USA	N/A	Binding analysis
ARLG-1912	USA	USA	N/A	Binding analysis
ARLG-1915	USA	USA	N/A	Binding analysis
C14	USA	USA	N/A	Binding analysis
C8	USA	USA	N/A	Binding analysis
HUMC1	USA	USA	SAMN14766402	Binding analysis, *in vivo* tests
HUMC10	USA	USA	N/A	Binding analysis
HUMC11	USA	USA	N/A	Binding analysis
HUMC12	USA	USA	N/A	Binding analysis
HUMC13	USA	USA	N/A	Binding analysis

HUMC14	USA	USA	N/A	Binding analysis
HUMC15	USA	USA	N/A	Binding analysis
HUMC16	USA	USA	N/A	Binding analysis
HUMC17	USA	USA	N/A	Binding analysis
HUMC18	USA	USA	N/A	Binding analysis
HUMC19	USA	USA	N/A	Binding analysis
HUMC2	USA	USA	N/A	Binding analysis
HUMC20	USA	USA	N/A	Binding analysis
HUMC21	USA	USA	N/A	Binding analysis
HUMC22	USA	USA	N/A	Binding analysis
HUMC23	USA	USA	N/A	Binding analysis
HUMC24	USA	USA	N/A	Binding analysis
HUMC3	USA	USA	N/A	Binding analysis
HUMC4	USA	USA	N/A	Binding analysis
HUMC5	USA	USA	N/A	Binding analysis, Immunization
HUMC6	USA	USA	N/A	Binding analysis
HUMC8	USA	USA	N/A	Binding analysis
HUMC9	USA	USA	N/A	Binding analysis
LAC-4	USA	USA	SAMN19068074	Binding analysis, *in vivo* tests
LACUSC-1	USA	USA	N/A	Binding analysis
Metro9	USA	USA	N/A	Binding analysis
R2	USA	USA	N/A	Binding analysis
UH034	USA	USA	N/A	Binding analysis
UH036	USA	USA	N/A	Binding analysis
UH042	USA	USA	N/A	Binding analysis
UH044	USA	USA	N/A	Binding analysis
UH045	USA	USA	N/A	Binding analysis
UH048	USA	USA	N/A	Binding analysis
UH049	USA	USA	N/A	Binding analysis
UH050	USA	USA	N/A	Binding analysis
UH051	USA	USA	N/A	Binding analysis
UH052	USA	USA	N/A	Binding analysis
UH060	USA	USA	N/A	Binding analysis
UH065	USA	USA	N/A	Binding analysis
UH070	USA	USA	N/A	Binding analysis
UH071	USA	USA	N/A	Binding analysis, Immunization
UH074	USA	USA	N/A	Binding analysis
UH075	USA	USA	N/A	Binding analysis
UH076	USA	USA	N/A	Binding analysis
UH080	USA	USA	N/A	Binding analysis
UH083	USA	USA	N/A	Binding analysis

UH086	USA	USA	N/A	Binding analysis
UH094	USA	USA	N/A	Binding analysis
UH096	USA	USA	N/A	Binding analysis
UH103	USA	USA	N/A	Binding analysis
UH108	USA	USA	N/A	Binding analysis
UH116	USA	USA	N/A	Binding analysis
UH117	USA	USA	N/A	Binding analysis
UH118	USA	USA	N/A	Binding analysis
UH122	USA	USA	N/A	Binding analysis
UH123	USA	USA	N/A	Binding analysis
UH125	USA	USA	N/A	Binding analysis
UH128	USA	USA	N/A	Binding analysis
UH132	USA	USA	N/A	Binding analysis
UH136	USA	USA	N/A	Binding analysis
UH143	USA	USA	N/A	Binding analysis
UH145	USA	USA	N/A	Binding analysis
UH152	USA	USA	N/A	Binding analysis
UH156	USA	USA	N/A	Binding analysis, *in vivo* tests
UH158	USA	USA	N/A	Binding analysis
UH186	USA	USA	N/A	Binding analysis
UH196	USA	USA	N/A	Binding analysis
UH197	USA	USA	N/A	Binding analysis
UH199	USA	USA	N/A	Binding analysis
UH203	USA	USA	N/A	Binding analysis
UH204	USA	USA	N/A	Binding analysis
UH205	USA	USA	N/A	Binding analysis
UH206	USA	USA	N/A	Binding analysis
UH208	USA	USA	N/A	Binding analysis
UH209	USA	USA	N/A	Binding analysis
UH210	USA	USA	N/A	Binding analysis
UH210	USA	USA	N/A	Binding analysis
UH211	USA	USA	N/A	Binding analysis
UH213	USA	USA	N/A	Binding analysis
UH214	USA	USA	N/A	Binding analysis
UH218	USA	USA	N/A	Binding analysis
UH219	USA	USA	N/A	Binding analysis
UH220	USA	USA	N/A	Binding analysis
UH2207	USA	USA	N/A	Binding analysis
UH223	USA	USA	N/A	Binding analysis
UH224	USA	USA	N/A	Binding analysis
UH267	USA	USA	N/A	Binding analysis

UH351	USA	USA	N/A	Binding analysis, *in vivo* tests
UH420	USA	USA	N/A	Binding analysis
UH4907	USA	USA	N/A	Binding analysis
UH5107	USA	USA	N/A	Binding analysis
UH516	USA	USA	N/A	Binding analysis
UH5207	USA	USA	N/A	Binding analysis
UH527	USA	USA	N/A	Binding analysis
UH569	USA	USA	N/A	Binding analysis
UH6507	USA	USA	N/A	Binding analysis
UH6907	USA	USA	N/A	Binding analysis
UH7007	USA	USA	N/A	Binding analysis
UH7507	USA	USA	N/A	Binding analysis
UH7607	USA	USA	N/A	Binding analysis
UH7807	USA	USA	N/A	Binding analysis
UH7907	USA	USA	N/A	Binding analysis
UH8107	USA	USA	N/A	Binding analysis
UH8307	USA	USA	N/A	Binding analysis
UH8407	USA	USA	N/A	Binding analysis
UH9007	USA	USA	N/A	Binding analysis
UH9707	USA	USA	N/A	Binding analysis
VA-Ab01	USA	USA	N/A	Binding analysis
VA-Ab02	USA	USA	N/A	Binding analysis
VA-Ab03	USA	USA	N/A	Binding analysis
VA-Ab06	USA	USA	N/A	Binding analysis
VA-Ab07	USA	USA	N/A	Binding analysis
VA-Ab08	USA	USA	N/A	Binding analysis
VA-Ab09	USA	USA	N/A	Binding analysis
VA-Ab10	USA	USA	N/A	Binding analysis
VA-Ab11	USA	USA	N/A	Binding analysis
VA-Ab12	USA	USA	N/A	Binding analysis
VA-Ab13	USA	USA	N/A	Binding analysis
VA-Ab14	USA	USA	N/A	Binding analysis
VA-Ab15	USA	USA	N/A	Binding analysis
VA-Ab16	USA	USA	N/A	Binding analysis
VA-Ab17	USA	USA	N/A	Binding analysis
VA-Ab18	USA	USA	N/A	Binding analysis
VA-Ab20	USA	USA	N/A	Binding analysis
VA-Ab22	USA	USA	N/A	Binding analysis
VA-Ab23	USA	USA	N/A	Binding analysis
VA-Ab29	USA	USA	N/A	Binding analysis
VA-Ab30	USA	USA	N/A	Binding analysis

VA-Ab32	USA	USA	N/A	Binding analysis
VA-Ab33	USA	USA	N/A	Binding analysis
VA-Ab36	USA	USA	N/A	Binding analysis
VA-Ab37	USA	USA	N/A	Binding analysis
VA-Ab39	USA	USA	N/A	Binding analysis
VA-Ab40	USA	USA	N/A	Binding analysis
VA-Ab41	USA	USA	N/A	Binding analysis, *in vivo* tests
VA-Ab42	USA	USA	N/A	Binding analysis
VA-Ab43	USA	USA	N/A	Binding analysis
VA-Ab44	USA	USA	N/A	Binding analysis
VA-Ab45	USA	USA	N/A	Binding analysis
VA-Ab51	USA	USA	N/A	Binding analysis
VA-Ab52	USA	USA	N/A	Binding analysis
VA-Ab53	USA	USA	N/A	Binding analysis
VA-Ab54	USA	USA	N/A	Binding analysis
VA-Ab55	USA	USA	N/A	Binding analysis
VA-Ab57	USA	USA	N/A	Binding analysis
VA-Ab58	USA	USA	N/A	Binding analysis
VA-Ab59	USA	USA	N/A	Binding analysis
VA-Ab60	USA	USA	N/A	Binding analysis
VA-Ab61	USA	USA	N/A	Binding analysis
VA-Ab62	USA	USA	N/A	Binding analysis
VA-Ab63	USA	USA	N/A	Binding analysis
VA-Ab64	USA	USA	N/A	Binding analysis
VA-Ab65	USA	USA	N/A	Binding analysis
VA-Ab66	USA	USA	N/A	Binding analysis, Immunization
VA-Ab67	USA	USA	N/A	Binding analysis
VA-Ab68	USA	USA	N/A	Binding analysis
VA-Ab69	USA	USA	N/A	Binding analysis
VA-Ab70	USA	USA	N/A	Binding analysis
VA-Ab71	USA	USA	N/A	Binding analysis
VA-Ab72	USA	USA	N/A	Binding analysis
VA-Ab73	USA	USA	N/A	Binding analysis
VA-Ab74	USA	USA	N/A	Binding analysis
VA-Ab75	USA	USA	N/A	Binding analysis
VA-Ab76	USA	USA	N/A	Binding analysis
VA-Ab77	USA	USA	N/A	Binding analysis
VA-Ab78	USA	USA	N/A	Binding analysis
VA-Ab79	USA	USA	N/A	Binding analysis
VA-Ab80	USA	USA	N/A	Binding analysis
VA-Ab81	USA	USA	N/A	Binding analysis

VA-Ab82	USA	USA	N/A	Binding analysis
VA-Ab83	USA	USA	N/A	Binding analysis
VA-Ab84	USA	USA	N/A	Binding analysis
VA-Ab85	USA	USA	N/A	Binding analysis
VA-Ab86	USA	USA	N/A	Binding analysis
VA-Ab87	USA	USA	N/A	Binding analysis
VA-Ab89	USA	USA	N/A	Binding analysis
VA-Ab90	USA	USA	N/A	Binding analysis
VA-Ab91	USA	USA	N/A	Binding analysis
VA-Ab92	USA	USA	N/A	Binding analysis
VA-Ab93	USA	USA	N/A	Binding analysis
VA-Ab94	USA	USA	N/A	Binding analysis
VA-Ab95	USA	USA	N/A	Binding analysis
VA-Ab96	USA	USA	N/A	Binding analysis
VA-Ab97	USA	USA	N/A	Binding analysis
VA-Ab98	USA	USA	N/A	Binding analysis
MRSN-10372	Diversity Panel	USA	SAMN12087724	K-type binding analysis
MRSN-11224	Diversity Panel	USA	SAMN12087725	K-type binding analysis
MRSN-11663	Diversity Panel	USA	SAMN12087726	K-type binding analysis
MRSN-11669	Diversity Panel	USA	SAMN12087727	K-type binding analysis
MRSN-11695	Diversity Panel	USA	SAMN12087728	K-type binding analysis
MRSN-11703	Diversity Panel	USA	SAMN12087729	K-type binding analysis
MRSN-1171	Diversity Panel	USA	SAMN12087697	K-type binding analysis
MRSN-1174	Diversity Panel	USA	SAMN12087698	K-type binding analysis
MRSN-11816	Diversity Panel	USA	SAMN12087730	K-type binding analysis
MRSN-1183	Diversity Panel	USA	SAMN12087699	K-type binding analysis
MRSN-1187	Diversity Panel	USA	SAMN12087700	K-type binding analysis
MRSN-1196	Diversity Panel	USA	SAMN12087701	K-type binding analysis, *in vivo* tests
MRSN-1311	Diversity Panel	USA	SAMN12087702	K-type binding analysis
MRSN-14193	Diversity Panel	Honduras	SAMN12087645	K-type binding analysis
MRSN-14237	Diversity Panel	Honduras	SAMN12087646	K-type binding analysis
MRSN-14427	Diversity Panel	Afghanistan	SAMN12087640	K-type binding analysis
MRSN-15049	Diversity Panel	Afghanistan	SAMN12087641	K-type binding analysis
MRSN-15070	Diversity Panel	USA	SAMN12087731	K-type binding analysis
MRSN-15075	Diversity Panel	USA	SAMN12087732	K-type binding analysis
MRSN-15088	Diversity Panel	USA	SAMN12087733	K-type binding analysis
MRSN-15093	Diversity Panel	Peru	SAMN12087688	K-type binding analysis
MRSN-15129	Diversity Panel	Peru	SAMN12087689	K-type binding analysis
MRSN-1551	Diversity Panel	USA	SAMN12087703	K-type binding analysis
MRSN-15574	Diversity Panel	Germany	SAMN12087653	K-type binding analysis
MRSN-16880	Diversity Panel	USA	SAMN12087734	K-type binding analysis

MRSN-17493	Diversity Panel	USA	SAMN12087735	K-type binding analysis, *in vivo* tests
MRSN-19482	Diversity Panel	Peru	SAMN12087690	K-type binding analysis
MRSN-21660	Diversity Panel	Honduras	SAMN12087647	K-type binding analysis
MRSN-21681	Diversity Panel	Honduras	SAMN12087648	K-type binding analysis
MRSN-22112	Diversity Panel	Peru	SAMN12087691	K-type binding analysis
MRSN-23390	Diversity Panel	Afghanistan	SAMN12087642	K-type binding analysis, *in vivo* tests
MRSN-24008	Diversity Panel	USA	SAMN12087736	K-type binding analysis
MRSN-24603	Diversity Panel	Germany	SAMN12087654	K-type binding analysis
MRSN-25547	Diversity Panel	Germany	SAMN12087655	K-type binding analysis
MRSN-2821	Diversity Panel	USA	SAMN12087704	K-type binding analysis
MRSN-29908	Diversity Panel	USA	SAMN12087737	K-type binding analysis
MRSN-29999	Diversity Panel	Germany	SAMN12087656	K-type binding analysis
MRSN-30000	Diversity Panel	Germany	SAMN12087657	K-type binding analysis
MRSN-30885	Diversity Panel	Germany	SAMN12087658	K-type binding analysis
MRSN-30896	Diversity Panel	Germany	SAMN12087659	K-type binding analysis
MRSN-30909	Diversity Panel	Germany	SAMN12087660	K-type binding analysis
MRSN-30912	Diversity Panel	Germany	SAMN12087661	K-type binding analysis
MRSN-30945	Diversity Panel	Germany	SAMN12087662	K-type binding analysis
MRSN-31159	Diversity Panel	Germany	SAMN12087663	K-type binding analysis
MRSN-31196	Diversity Panel	Germany	SAMN12087664	K-type binding analysis
MRSN-31461	Diversity Panel	Germany	SAMN12087665	K-type binding analysis
MRSN-31468	Diversity Panel	Germany	SAMN12087666	K-type binding analysis
MRSN-31523	Diversity Panel	Germany	SAMN12087667	K-type binding analysis
MRSN-31915	Diversity Panel	Germany	SAMN12087668	K-type binding analysis
MRSN-31937	Diversity Panel	Germany	SAMN12087669	K-type binding analysis
MRSN-31942	Diversity Panel	Germany	SAMN12087670	K-type binding analysis
MRSN-31947	Diversity Panel	Germany	SAMN12087671	K-type binding analysis
MRSN-32076	Diversity Panel	Germany	SAMN12087672	K-type binding analysis
MRSN-32104	Diversity Panel	Germany	SAMN12087673	K-type binding analysis
MRSN-32108	Diversity Panel	Germany	SAMN12087674	K-type binding analysis
MRSN-32142	Diversity Panel	Germany	SAMN12087675	K-type binding analysis
MRSN-32304	Diversity Panel	USA	SAMN12087738	K-type binding analysis
MRSN-32797	Diversity Panel	Germany	SAMN12087676	K-type binding analysis
MRSN-32842	Diversity Panel	Germany	SAMN12087677	K-type binding analysis
MRSN-32865	Diversity Panel	Germany	SAMN12087678	K-type binding analysis
MRSN-32866	Diversity Panel	Germany	SAMN12087679	K-type binding analysis
MRSN-32875	Diversity Panel	Germany	SAMN12087680	K-type binding analysis
MRSN-32892	Diversity Panel	Germany	SAMN12087681	K-type binding analysis
MRSN-32915	Diversity Panel	Germany	SAMN12087682	K-type binding analysis
MRSN-334	Diversity Panel	USA	SAMN12087692	K-type binding analysis

MRSN-3360	Diversity Panel	USA	SAMN12087705	K-type binding analysis
MRSN-337038	Diversity Panel	Germany	SAMN12087683	K-type binding analysis
MRSN-351162	Diversity Panel	Germany	SAMN12087684	K-type binding analysis
MRSN-351524	Diversity Panel	Germany	SAMN12087685	K-type binding analysis
MRSN-3658	Diversity Panel	Germany	SAMN12087649	K-type binding analysis, in vivo tests
MRSN-3692	Diversity Panel	Germany	SAMN12087650	K-type binding analysis
MRSN-3874	Diversity Panel	Germany	SAMN12087651	K-type binding analysis
MRSN-423159	Diversity Panel	USA	SAMN12087739	K-type binding analysis
MRSN-4484	Diversity Panel	USA	SAMN12087706	K-type binding analysis
MRSN-480561	Diversity Panel	Afghanistan	SAMN12087643	K-type binding analysis
MRSN-480622	Diversity Panel	Afghanistan	SAMN12087644	K-type binding analysis
MRSN-489669	Diversity Panel	Ukraine	SAMN12087686	K-type binding analysis
MRSN-489678	Diversity Panel	Ukraine	SAMN12087687	K-type binding analysis
MRSN-4943	Diversity Panel	USA	SAMN12087707	K-type binding analysis
MRSN-5969	Diversity Panel	Germany	SAMN12087652	K-type binding analysis
MRSN-6541	Diversity Panel	USA	SAMN12087708	K-type binding analysis
MRSN-7067	Diversity Panel	USA	SAMN12087709	K-type binding analysis
MRSN-7113	Diversity Panel	USA	SAMN12087710	K-type binding analysis
MRSN-7124	Diversity Panel	USA	SAMN12087711	K-type binding analysis
MRSN-7137	Diversity Panel	USA	SAMN12087712	K-type binding analysis
MRSN-7153	Diversity Panel	USA	SAMN12087713	K-type binding analysis
MRSN-7213	Diversity Panel	USA	SAMN12087714	K-type binding analysis
MRSN-7251	Diversity Panel	USA	SAMN12087715	K-type binding analysis
MRSN-7431	Diversity Panel	USA	SAMN12087716	K-type binding analysis
MRSN-7446	Diversity Panel	USA	SAMN12087717	K-type binding analysis
MRSN-7460	Diversity Panel	USA	SAMN12087718	K-type binding analysis
MRSN-7521	Diversity Panel	USA	SAMN12087719	K-type binding analysis, in vivo tests
MRSN-7576	Diversity Panel	USA	SAMN12087720	K-type binding analysis
MRSN-7690	Diversity Panel	USA	SAMN12087721	K-type binding analysis
MRSN-7725	Diversity Panel	USA	SAMN12087722	K-type binding analysis
MRSN-7735	Diversity Panel	USA	SAMN12087723	K-type binding analysis
MRSN-843	Diversity Panel	USA	SAMN12087693	K-type binding analysis
MRSN-918	Diversity Panel	USA	SAMN12087694	K-type binding analysis
MRSN-959	Diversity Panel	USA	SAMN12087695	K-type binding analysis
MRSN-960	Diversity Panel	USA	SAMN12087696	K-type binding analysis

Table S4. Summary of strains used in each study. Table shows briefly each strain used, the panel it belonged to, its nation of origin, what it was used for, as well as its accession number where available.
